# A Review on Low-Grade Thermal Energy Harvesting: Materials, Methods and Devices

**DOI:** 10.3390/ma11081433

**Published:** 2018-08-14

**Authors:** Ravi Anant Kishore, Shashank Priya

**Affiliations:** 1 Center for Energy Harvesting Materials and Systems, Virginia Polytechnic Institute and State University, Blacksburg, VA 24061, USA; ravi86@vt.edu; 2 Materials Research Institute, Pennsylvania State University, University Park, PA 16802, USA

**Keywords:** thermoelectric, pyroelectric, thermomagnetic, thermoelastic

## Abstract

Combined rejected and naturally available heat constitute an enormous energy resource that remains mostly untapped. Thermal energy harvesting can provide a cost-effective and reliable way to convert available heat into mechanical motion or electricity. This extensive review analyzes the literature covering broad topical areas under solid-state low temperature thermal energy harvesting. These topics include thermoelectricity, pyroelectricity, thermomagneticity, and thermoelasticity. For each topical area, a detailed discussion is provided comprising of basic physics, working principle, performance characteristics, state-of-the-art materials, and current generation devices. Technical advancements reported in the literature are utilized to analyze the performance, identify the challenges, and provide guidance for material and mechanism selection. The review provides a detailed analysis of advantages and disadvantages of each energy harvesting mechanism, which will provide guidance towards designing a hybrid thermal energy harvester that can overcome various limitations of the individual mechanism.

## 1. Introduction

The ever-growing energy demand, escalating energy prices, and environmental concerns such as global warming compel us to look for cleaner and more sustainable energy sources. Thermal energy harvesting is a promising method for capturing freely available heat and converting it to a more usable form, such as mechanical or electrical energy. The ‘free’ heat is available to us primarily in two forms: waste heat and nature heat. It has been reported that more than half of the energy produced from various renewable and non-renewable resources worldwide is rejected to the environment, mostly in the form of waste heat [1]. In addition, natural resources such as geothermal heat, volcanic heat, and solar heat are the enormous energy resources that remain untapped. Plenty of such thermal energy reserves exist all over the world, which release thousands of joules of energy every second into the ambient atmosphere [2]. Unarguably, a cost-effective method for recovering waste heat and utilizing natural heat to generate electricity can revolutionize the production of renewable energy. Table 1 shows some of the major waste heat sources along with the temperature range. Based upon the temperature, heat is usually classified into three categories: high-grade (1200 °F/649 °C and higher), medium-grade (450 °F/232 °C to 1200 °F/649 °C), and low-grade (450 °F/232 °C and lower) [3]. Normally high and medium grade heat is easy to recover; whereas low-grade waste heat, which constitutes more than 50% of the total waste heat, is unfortunately the most difficult to recover [3]. Considering hot and cold reservoirs at 232 °C and 25 °C respectively, it can be calculated that the Carnot efficiency of a low-grade heat recovery engine cannot be more than 41%. The Carnot efficiency decreases to 24% when the operating temperature is between 150 °C and 50 °C. Such low efficiency makes the recovery process economically unviable using traditional power cycles.

Steam Rankine cycle based power plants are currently the most common technology used to obtain work from heat. Steam Rankine cycle is simple to implement as heat is utilized to generate steam, which is eventually used to drive steam turbine. The traditional steam Rankine cycle has been recommended as the most efficient option for high- and medium-grade waste heat recovery [5]. For low-grade heat recovery, however, steam Rankine cycles are not cost-effective, as low-pressure and low-temperature steam requires bulkier equipment and produces water condensates on turbine blades causing the erosion [3]. For medium-to-low temperature operation, Rankine cycle requires a working fluid that has a lower boiling point than water. Examples of such fluids include silicon oil, propane, haloalkanes, isopentane, iso­butane, p­xylene, and toluene [3]. As it can be noticed, most of these fluids are organic compounds and therefore the modified thermodynamic cycle is traditionally referred to as the Organic Rankine cycle (ORC). The working principle of ORC is the same as the traditional Rankine cycle, except it utilizes organic fluids instead of steam. Compared with the steam Rankine cycle, ORC usually has simple structure, high reliability, and low maintenance cost, since its uses organic fluids having low boiling temperatures [6,7]. ORC has been proposed for waste heat recovery from a variety of areas including geothermal [8,9], solar [10,11], gas turbine [12], and combustion engines [13,14]. However, ORC presents several challenges and the most important one is finding a suitable organic fluid. Studies have shown that there is no single working fluid that is optimal for all ORC applications [15]. In addition, the overall efficiency of ORC based power plant has been reported to be in the range of 10–20%, which is much lower than the efficiency (30–40%) of the high temperature steam Rankine cycle [3]. Another alternative for a medium-to-low temperature heat source is the Kalina cycle. Unlike the steam and organic Rankine cycles that employ a single fluid, the Kalina cycle uses a binary fluid (i.e., solution of two fluids with different boiling points, such as ammonia and water) as the working fluid. The temperature of a single­fluid system remains constant during boiling and condensation. In contrast, the temperature of a binary mixture system (containing two fluids of different boiling point) increases during evaporation. This allows binary fluid to obtain a better thermal matching with the heat source in the evaporator and with the cooling medium in the condenser [3], enabling the Kalina cycle to achieve energy conversion efficiency close to the efficiency of the steam Rankine cycle [16]. The Kalina cycle, however, has some drawbacks. First, the Kalina cycle requires a complicated plant scheme [17]. Second, high vapor fraction in the boiler results in low heat transfer coefficients, and thus a large heat exchanger is required. The other drawback of the Kalina cycle is a relatively higher corrosion rate. Impurities such as air or carbon dioxide in liquid ammonia cause stress corrosion cracking in fluid carrying mild-steel hydraulic pipes. Ammonia is also highly corrosive towards copper and zinc [18]. Lastly, the Kalina cycle is a proprietary technology. All first generation global patents related to the Kalina cycle processes are owned by Wasabi Energy [19] and Kalex LLC owns licensing the rights for deploying the second generation Kalina cycle [20]. Considering these limitations and the practical challenges, the Kalina cycle is not always justifiable, especially for small-scale low-grade heat recovery [21].

**Figure 1:**
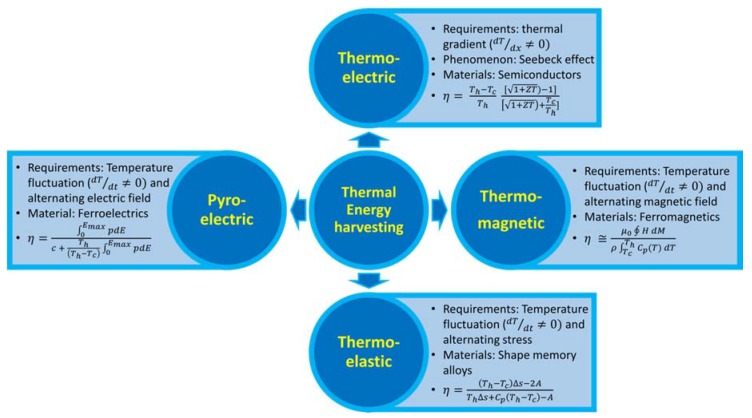
Relevant thermal energy harvesting technologies: thermoelectricity, pyroelectricity, thermomagneticity, and thermoelasticity. Thermal energy harvesters are solid-state devices that do not require any fuel and do not produce harmful discharge.

In the last few decades, immense efforts have been made to explore and develop alternative technologies to capture low-grade heat. The low-grade thermal energy harvesting technologies are currently based upon the thermoelectric, pyroelectric, thermomagnetic, and thermoelastic effect (Figure 1). Among these, thermoelectric energy harvesting is the most popular and studied technology. Thermoelectric generators (TEGs) are based upon the Seebeck effect and they generate direct current in response to thermal gradients. Pyroelectric, thermomagnetic, and thermoelastic energy harvesting methods are also old concepts, but practical developments in these fields has been quite sluggish in comparison to thermoelectric devices, primarily because of their requirement of temperature fluctuation. Electric, magnetic, or elastic properties of certain materials change due to fluctuation in temperature, and this phenomenon is used to produce mechanical or electrical work.

There are several advantages in using thermal energy harvesters over traditional heat engines. The thermal energy harvesters incur minimal operational cost as they utilize freely available thermal energy such as waste heat and natural heat and convert it in to useful work. In comparison to traditional heat engines, thermal energy harvesters operate at low temperature and therefore have suitability for applications such as powering sensor nodes. Thermal energy harvesters are solid-state devices, do not produce harmful discharge, and have very low maintenance cost. They possess low energy conversion efficiency, typically 1–10%, but are self-sustainable, which makes them suitable for providing power in remote or hard-to–reach areas. The reliability of these devices can be understood from the fact that NASA uses Radioisotope Thermoelectric Generators (RTGs) for powering the spacecraft. One of the available report states that RTGs have been used for a total combined time of over 300 years, and a not a single thermocouple has ever ceased producing power [22].

**Figure 2:**
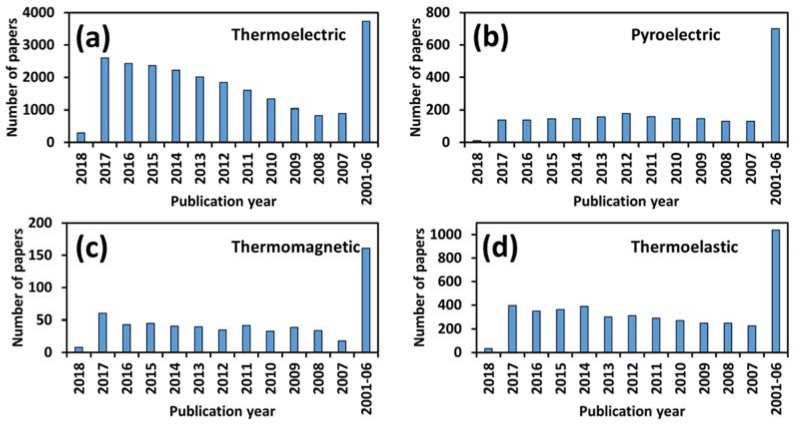
Number of scientific papers published in the field of (**a**) thermoelectric, (**b**) pyroelectric, (**c**) thermomagnetic, and (**d**) thermoelastic effect from the year 2001 to present. These numbers were obtained by searching phrases “thermoelectric”, “pyroelectric”, “thermomagnetic”, and “thermoelastic” in the title of the articles on Google scholar. The actual publication on research in these fields might be higher than the values presented here.

Figure 2 shows the number of scientific papers published in the field of thermoelectric, pyroelectric, thermomagnetic, and thermoelastic from the year 2001 to present. It can be noted that, collectively, about 3200 papers were published in 2017 and looking at the trend, this number is expected to increase in the forthcoming years. Undoubtedly, thermal energy harvesting is a very hotly pursued subject undergoing intense investigation by the scientific community. While the focus of many of these studies is to develop an efficient material, there is parallel emphasis on developing methodology to optimize the system level performance. A review on this topic that summarizes important technical advancements and then utilizes the published information to analyze the performance, identify the challenges, and provide guidance for material and mechanism selection will serve the community to focus its energy on addressing the relevant challenges. This review provides a comprehensive summary of materials, designs, models, and experimental results reported in literature and analyzes the information to provide guidance for the development of thermal energy harvesters. To our knowledge, this is the first review paper where various facets of thermal energy harvesting have been analyzed together. While each thermal harvesting methodology has certain advantages and disadvantages, a combinatory study is necessary to shed new light on understanding the differences, which will help researchers to integrate the information in systematic fashion. The information presented in this study has been obtained after an exhaustive literature review, comprising of more than 300 journal papers. While our focus is to provide the most recent findings, we have also attempted to briefly include the historical developments. Broadly, the study provides a discussion on basic physics, working principle, performance measurements, materials, and current state-of-the-art devices.

## 2. Thermoelectric Energy Harvesting

Thermoelectric (TE) effect is the resultant of four different phenomena: Joule heating, Seebeck effect, Peltier effect, and Thomson effect [23]. Joule heating, also termed as resistive heating, occurs when an electric current flows through an electrical conductor. Joule or resistive heat is directly proportional to square of the current and the electrical resistance of the conductor. The other three effects are described in the following sections.

### 2.1. Seebeck Effect

**Figure 3:**
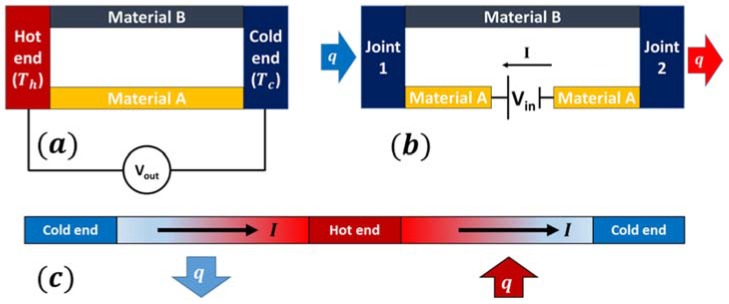
(**a**) Seebeck effect: Two thermoelectric materials joined at hot and cold junctions, which are maintained at temperatures $T_{h}$ and $T_{c}$ (temperature difference $\Delta T=T_{h}-T_{c}$). Seebeck EMF of $V_{out}$ is obtained across the junctions; (**b**) Peltier effect: Two thermoelectric materials joined at the two ends. An electric current ‘I’ is passed through the circuit and heat q is evolved at one end and absorbed at the other; (**c**) Thomson effect: due to spatial gradient in temperature and current, a continuous version of the Peltier effect occurs, resulting in Thomson heat.

When two dissimilar electrical conductors or semiconductors are joined together, they make a thermocouple and if the temperature difference is maintained between the two joining junctions, an electromotive force (EMF) is developed (Figure 3a). This phenomenon is called the Seebeck effect and it was first observed by Thomas Johann Seebeck in 1821. This induced voltage is called Seebeck EMF and it is directly proportional to the temperature difference. If $V_{out}$ is the obtained voltage difference, ∆*T* is the temperature difference, then the Seebeck coefficient, α (also known as thermopower, thermoelectric power, and thermoelectric sensitivity) is given as [24,25]

(1) $$\alpha_{ab}=\frac{V_{out}}{∆T}$$

where subscript ab signifies two conductors, A and B, of the thermocouple. All materials exhibit the TE effect, to some extent. The known materials typically have a Seebeck coefficient between 100 and 1000 µV/K. Metal such as iron has a Seebeck coefficient of around 19 μV/°C at 0 °C. Some materials can also have a negative Seebeck coefficient. For example, constantan has a Seebeck coefficient of −35 μV/°C at 0 °C. It is important to note that these specified values of the Seebeck coefficient are at 0 °C. The Seebeck coefficient is a highly temperature dependent parameter and its value varies non-linearly with temperature. Most metals exhibit Seebeck coefficients of 10 μV/°C or less. However, semiconductors have Seebeck coefficients higher than 100 μV/°C; therefore, they are commonly used in thermoelectric modules. Semiconductors having excess electrons show negative a Seebeck coefficient and are called a n-type semiconductor. On the other hand, semiconductors having excess holes exhibit a positive Seebeck coefficient and are called a p-type semiconductor. The effective Seebeck coefficient of a thermoelectric couple consisting of one p-type and one n-type semiconductor elements is given as:

(2) $$\alpha_{pn}=\alpha_{p}-\alpha_{n}$$

### 2.2. Peltier Effect

The Peltier effect is reverse of the Seebeck effect and was first observed by Jean Charles Athanase Peltier in 1834. The Peltier effect results in cooling or heating at the junction of two dissimilar thermoelectric (TE) materials when an electric current is passed through the junction (Figure 3b). The magnitude of Peltier heat is directly proportional to the current but its sign (cooling or heating effect) depends on the direction of the electric current. The rate of evolved or absorbed heat, q, is directly proportional to the electric current in the circuit and is given as [26]:

(3) $$q=\left(\pi_{a}-\pi_{b}\right)I$$

where *I* denotes the electric current from conductor A to B and $\pi_{a}$ and $\pi_{b}$ are the proportionality constant known as Peltier coefficient for conductors A and B.

### 2.3. Thomson Effect

Thomson effect occurs in a non-uniformly heated TE material. For most TE materials, Seebeck coefficient varies with temperature. Therefore, when a spatial temperature gradient exists in a TE material and current is passed through it, a continuous version of the Seebeck and Peltier effects occur, resulting in Thomson heat (Figure 3c). Thomson heat is absorbed or released depending upon the direction of current, but its magnitude is proportional to the magnitude of the current. The rate of heating per unit length of the conductor is directly proportional to the electric current and temperature gradient [25]:

(4) $$\frac{dq}{ds}=\tau I\frac{dT}{ds}$$

where τ is defined as the Thomson coefficient and *s* is a spatial coordinate.

It is important to note that the Seebeck and Peltier effects occur with a thermocouple, whereas Thomson effect exists for a single conductor. In addition, Seebeck coefficient, α, Peltier coefficient, π, and Thomson coefficient, μ, are related using Kelvin relations [23]:

(5) $$\pi_{ab}=\alpha_{ab}T$$

(6) $$\tau_{a}-\tau_{b}=T\frac{d\alpha_{ab}}{dT}$$

where $\alpha_{ab}=\alpha_{a}-\alpha_{b}$ and

(7) $$\pi_{ab}=\pi_{a}-\pi_{b}$$

### 2.4. Working Principle

**Figure 4:**
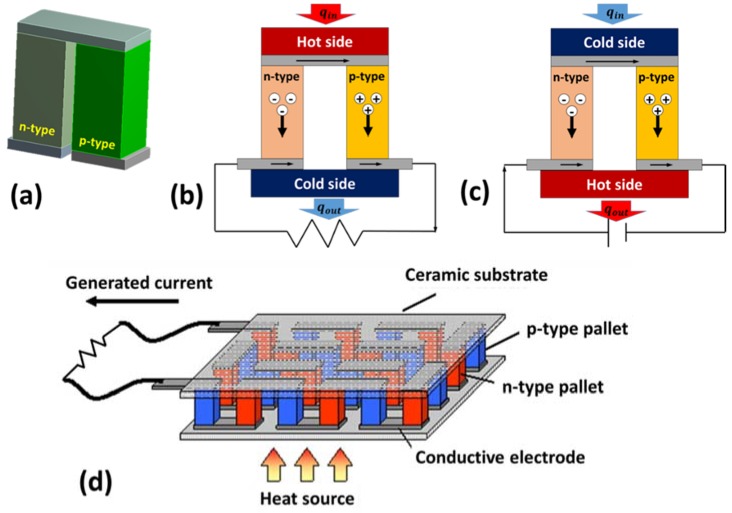
(**a**) A thermocouple consisting of one p-type and one n-type semiconductor pellets. The p-type leg contains excess holes, whereas the n-type leg contains excess electrons; (**b**) Mechanism of thermoelectric power generation. Positive charge flow from the p-type to n-type leg in the external circuit resulting in electric current; (**c**) Mechanism of thermoelectric refrigeration. External current makes charge carriers to carry heat from cold-side to hot-side; (**d**) Key components of a thermoelectric generator (TEG) module [31].

A combination of one p-type and one n-type semiconductor material in rigid form, such as pellets, connected electrically in series and thermally in parallel forms a thermocouple. As shown in Figure 4a, thermocouples are usually π-shaped. The two pellets making the thermocouple are called the legs of the thermocouple. Figure 4b, shows the thermocouple’s operation in power generation mode. The thermocouple is connected to an external electric circuit and a thermal gradient is applied across the two sides. The p-type semiconducting leg is dominated by holes as a charge carrier, whereas the n-type semiconducting leg has electrons as a majority carrier. As indicated in Figure 4b, holes and electrons travel from the hot side to the cold side in p-type and n-type legs, respectively, resulting in an electric current flowing in the external circuit from the p-type leg to the n-type leg. Though it is not the focus of this paper, the working mechanism of a thermoelectric cooler (TEC) is shown in Figure 4c. A voltage source is connected across the two free ends of the thermocouple. The external electric current forces the charge carriers to carry heat from the cold-side to the hot-side, as shown in Figure 4c. Figure 4d depicts key components of a thermoelectric generator (TEG) module. A TEG consists of several thermocouples connected together using conductive metal electrodes. In order to prevent inter-diffusion between the TE materials and electrode materials and to reduce contact resistance, diffusion barrier layers of Ti [27], Mo [28], CrSi, or Ni [29] are normally used at the interface [30]. The arrays of thermocouples are then mounted between thin ceramic substrates, such as Al_2_O_3_ (Alumina) and AlN (Aluminum Nitride), to electrically insulate the system.

### 2.5. Thermoelectric Properties

#### 2.5.1. Figure of Merit

**Figure 5:**
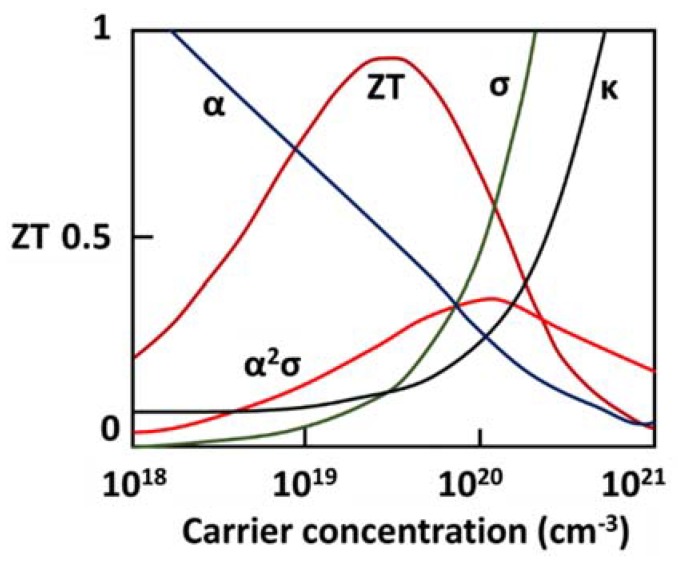
Variation of transport parameters: α, σ, and k, figure of merit, ZT, power factor, *α*^2^*σ*, as a function of carrier concentration for bismuth telluride. Figure redrawn from reference [33]. The electrical and thermal conductivities, σ and k, increase with an increase in carrier concentration, while the Seebeck coefficient, α, decreases with an increase in carrier concentration. This indicates that maximizing ZT requires optimization of α, σ, and k, simultaneously.

The thermoelectric performance of a given material is normally measured using a dimensionless figure of merit (ZT), which is defined as [32]:

(8) $$\mathrm{ZT}=\frac{\alpha^{2}\sigma }{k}T$$

where α is Seebeck coefficient, σ is electrical conductivity, k is thermal conductivity, and T is temperature in Kelvin. A higher ZT value of a TE material implies a higher thermoelectric effect, which usually leads to better performance by a TE device. Normally, it is expected that a TE material should have a large Seebeck coefficient, *α*, high electrical conductivity, *σ*, and low thermal conductivity, k. However, the three transport parameters: α, σ, and k are not independent and depend upon a variety of factors such as band structure and carrier concentration. Figure 5 illustrates the variation of these parameters with carrier concentration for bismuth telluride, one of the most common TE material [33]. It can be noted that both electrical and thermal conductivities, σ and k, increase with an increase in carrier concentration. Seebeck coefficient, α, has an opposite trend compared to σ and k. This indicates that figure of merit, ZT, cannot be improved by continuously increasing the carrier concentration, rather maximizing ZT requires optimizing α, σ, and k, simultaneously.

#### 2.5.2. Power Factor

As indicated by Equation (8), the figure of merit can be optimized by maximizing the electrical conductivity and Seebeck coefficient and by minimizing thermal conductivity. Electrical conductivity and Seebeck coefficient of a TE material are determined only by its electronic properties; therefore, both these parameters can be combined to define a term called power factor (*pf*), which is given as [34]:

(9) $$pf=\sigma \alpha^{2}$$

It can be observed from Figure 4 that similar to ZT, the maximum pf occurs at an optimal value of σ and α. However, pf maximizes at a higher value of carrier concentration than ZT. Assuming the single parabolic band and acoustic phonon scattering, the Seebeck coefficient in metals or degenerate semiconductors is given by [33]:

(10) α=8π2kB23eh2m∗T(π3n)23

where n is the carrier concentration, $m^{*}$ is the effective mass of the carrier, $k_{B}$ is the Boltzmann constant, h is the Planck constant, and e is the elementary charge. It can be noted from Equation (10) that Seebeck coefficient is inversely proportional to carrier concentration. This implies that insulators, having low carrier concentration, has a large Seebeck coefficient. The Seebeck coefficient can be further improved by reducing the carrier concentration. However, it adversely affects electrical conductivity, which is given as [33]

(11) σ=neμ

where μ is carrier mobility.

#### 2.5.3. Thermal Conductivity

The total thermal conductivity, k, in a TE material is the resultant of two contributions: (1) carriers: electrons and holes transporting heat ($k_{e}$) and (2) phonons traveling through the lattice ($k_{L}$) [35].

(12) $$k=k_{e}+k_{L}$$

The electronic thermal conductivity is directly related to the electrical conductivity through the Wiedemann–Franz law and it increases with an increase in carrier concentration [33].

(13) $$k_{e}=L\sigma T=ne\mu LT$$

where *L* is the Lorenz factor for free electrons.

In addition to electrons and holes transporting heat, heat transport in TE materials occurs by way of elastic vibrations of the lattice, called phonons. This transport mode is determined by the elastic scattering of acoustic phonons at lattice defects and the value of $k_{L}$ depends on the structure, rigidity, atomic masses, and other characteristics of the lattice [36]. In order to improve ZT of a TE material, its $k_{L}$ needs to be reduced.

#### 2.5.4. Improving ZT Value

**Figure 6:**
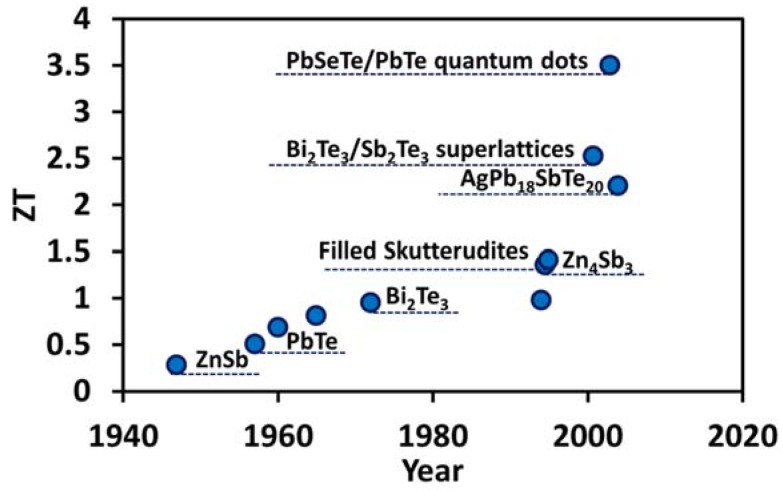
Key historical developments in figure of merit (ZT) of thermoelectric materials over the last century. ZT value for commercial TE materials are less than unity. Nanostructured ultra-thin film TE materials have shown ZT up to 3.5. Figure drawn using information in references [37,38,39].

Thermoelectric effect has been studied for more than a century. Despite many decades of effort, ZT of the commercialized thermoelectric materials, such as bismuth telluride (Bi_2_Te_3_) is still close to unity. For most bulk materials, electrical conductivity and thermal conductivity are directly related whereas electrical conductivity and the Seebeck coefficient are inversely related. This makes it very difficult to increase electrical conductivity and Seebeck coefficient together while decreasing the thermal conductivity at the same time. Figure 6 depicts the key historical developments in ZT of TE materials over the last century [37,38,39]. Some early attempts in the 1990s resulted in TE materials, such as Zn_4_Sb_3_-based materials (375–675 K) and Ce-based filled skutterudite (675–975 K), with ZT value over one. More recently, few state-of-the-art TE materials reported in the literature are quantum-dot superlattice with ZT of 3.5 at 575 K by Harman et al. [40], thin film superlattice structure with ZT of 2.4 at 300 K and ZT of 2.9 at 400 K by Venkatasubramanian et al. [41], and lead antimony silver telluride (AgPb_m_SbTe_2+m_) with ZT of 2.2 at 800 K by Hsu et al. [42]. These improvements in ZT are primarily due to a reduction in lattice thermal conductivity. There are broadly three approaches to reduce lattice thermal conductivity:

(1) Phonon scattering: Any mechanism that scatters phonons more effectively than the electrons or holes reduces the phonons’ mean free path, and thus enhances the electrical to thermal conductivity ratio of a TE material. Some of the main scattering mechanisms are scattering of phonons by phonons, scattering of phonons by grain boundaries, scattering of phonons by lattice defects/impurity, and scattering of phonons by electrons and holes. Detailed information on this can be found in reference [43].
(2) Complex crystal structures: Complex crystal structures are used to separate the electron-crystal region from the phonon-glass region, so that the region responsible for electron transport should be an electron crystal of a high-mobility semiconductor, whereas the phonon glass should contain the disordered structures and dopants. In 1995, Slack [43] proposed the concept of phonon-glass electron-crystal (PGEC) that would possess electronic conductivity similar to that of a single crystal semiconductor but would have thermal conductivity similar to that of an amorphous material. Later, this concept culminated in discovery of two distinct families of TE materials: filled skutterudites [44,45,46] and intermetallic clathrates [47,48,49].
(3) Nanostructuring: Most of the recent advancements in enhancing ZT is associated with nanostructuring. Because of the quantum size effects on energy carriers, it has been observed that the thermal conductivity of nanostructures such as superlattices are significantly lower than that of the bulk constituent materials [50]. This results in considerable improvement in figure-of-merit in superlattice systems. The concept of superlattices was first introduced by Dresselhaus, Harman, and Venkatasubramanian [51], and since then it has been extensively studied to understand the mechanism for improvement of electron performance, such as electron energy filtering [52], thermionic emission [53], and reduction of phonon thermal conductivity through interface scattering [54]. As shown in Figure 6, Bi_2_Te_3_/Sb_2_Te_3_ based superlattices [41] and PbTe-based quantum dot super-lattices [55] are currently the state-of-art TE materials.

### 2.6. Thermoelectric Materials

**Figure 7:**
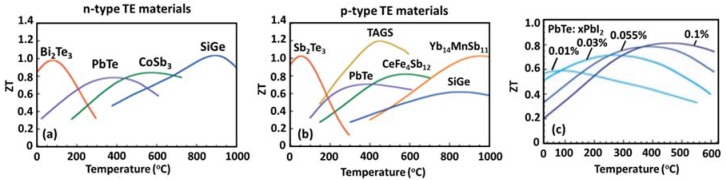
Some of the widely used TE materials with their operating temperature and figure-of-merit, ZT. (**a**) n-type TE materials; (**b**) p-type TE materials. Most of the TE materials shown here are complex alloys with dopants and compositions approximated; (**c**) Variation in peak ZT and the optimal temperature with change in dopant concentration for n-type PbTe. Changing the dopant concentration not only alters the peak ZT but also the optimal temperature where the peak occurs. Adapted from [33], with permission from © 2008 Springer Nature.

There are many families of TE materials developed for a wide range of applications targeting different operating temperature ranges. Some of them are shown in Figure 7. For low temperature (less than 500 K) applications, the alloys of Bi_2_Te_3_ and Sb_2_Te_3_ are widely used. ZT can be optimized in a desired temperature range by adjusting the carrier concentration through material composition in p-type alloy of form Bi_2−x_Sb_x_Te_3_ and n-type alloy of form Bi_2_Te_3−y_Se_y_. Studies have revealed that p-type compositions near (Sb_0.8_Bi_0.2_)_2_Te_3_ and n-type compositions close to Bi_2_(Te_0.8_Se_0.2_)_3_ provide the highest ZT, ~1.1 for p-type and ~0.8 for n-type [33]. However, a recent study has reported peak ZT of 1.4 at 100 °C for a p-type nanocrystalline BiSbTe bulk alloy, which were manufactured by ball milling followed by direct-current hot pressing under inert conditions [56]. For applications below room temperature, alloys of BiSb have been suggested [57,58].

TE materials based on group-IV tellurides, such as PbTe [59], GeTe [60,61,62], and SnTe [63,64,65] are normally suggested for mid-temperature range application (500–900 K) [66,67]. Among them, PbTe is the most widely used TE material and has maximum ZT in range of 0.8–1.8 above 700 K. PbTe can be doped with appropriate dopants, such as I and Tl [68,69,70], in order to tune the optimal temperature and enhance the ZT value. The effect is shown in Figure 7c. Some of the other state-of-art mid-temperature TE materials reported in the literature are skutterudite alloys of CoSb_3_ and CeFe_4_Sb_12_ with peak ZT ~0.8, TAGS ((AgSbTe_2_)_0.15_(GeTe)_0.85_ with peak ZT ~1.2 at 720 K [67]), Zn_4_Sb_3_ with peak ZT ~1.3 at 670 K [71]), Ag(Pb_1−y_Sn_y_)_m_SbTe_2+m_ series exhibiting peak ZT ~1.45 at 630 K [72], and AgPb_m_SbTe_2+m_ series with peak ZT ~2.2 at 800 K [42].

**Figure 8:**
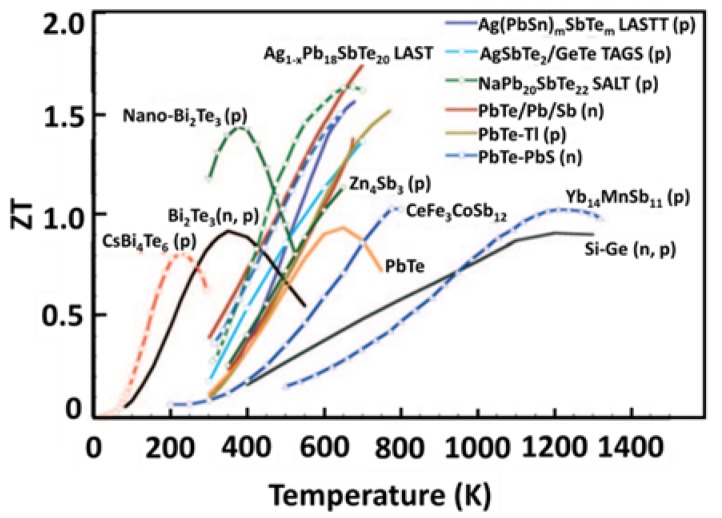
ZT plot of some of the state-of-the-art thermoelectric materials. Adapted from [73], with permission from © 2009 John Wiley and Sons Inc.

Silicon–germanium alloys (SiGe) are mostly used for high-temperature (>900 K) thermoelectric applications. The ZT value of these TE materials is less than one as shown in Figure 7, because of the relatively high lattice thermal conductivity of the diamond structure [33]. Figure 8 shows the ZT plot of some additional thermoelectric materials [73].

### 2.7. Thermoelectric Efficiency

Figure of merit is a measure of material performance. However, the performance of an energy-producing device, such as a thermoelectric generator, needs to be measured in terms of its power output and energy conversion efficiency. Thermoelectric effect is a complex phenomenon consisting of three different effects. Therefore, a complete mathematical formulation of TEGs requires using advanced mathematics and physics covering three-dimensional formulations [74,75,76,77]. For brevity, in this review, we will discuss the simplified one-dimensional model. Any energy loss, such as due to electrical and thermal contact resistances and due to convection and radiation from the faces of thermocouples, will be neglected. In order to explain the modeling procedure, we will derive the equation for just one thermocouple; however, formulation can be easily extended for a complete TEG module consisting of multiple thermocouples.

Let us consider a thermocouple connected to an external resistive load, *RL*, as shown in Figure 3b. Assuming constant thermoelectric properties (α, *σ*, and *k*), the steady state heat transfer rate $q_{h}$ (heat absorbed) on the hot side and $q_{c}$ (heat evolved) on the cold side can be expressed as [43,78]:

(14) $$q_{h}=\alpha_{pn}IT_{h}+k\left(T_{h}-T_{c}\right)-\frac{1}{2}I^{2}R$$

(15) $$q_{c}=\alpha_{pn}IT_{c}+k\left(T_{h}-T_{c}\right)+\frac{1}{2}I^{2}R$$

where effective Seebeck coefficient $\alpha_{pn}=\alpha_{p}-\alpha_{n}$, effective internal resistance $R=\frac{L}{\sigma_{p}A_{p}}+\frac{L}{\sigma_{n}A_{n}}$, and effective thermal conductance $k=\frac{1}{L}\left(k_{p}A_{p}+k_{n}A_{n}\right)$. $A_{p}$ and $A_{n}$ are the cross-sectional area of p and n legs, respectively, and L represents their length.

In Equations (14) and (15), the first terms represent Peltier heat (power generated), the second terms denote Fourier heat transfer, and the third terms denote Joule heat. The factor (1/2) for the Joule term is necessary to indicate that the total generated Joule heat is equally consumed between hot and cold sides, since TEG modules have an equal number of p-type and n-type elements [79].

The current, *I*, in the thermocouple equals the Seebeck EMF over total resistance (internal resistance *R* and external resistive load *RL*).

(16) $$I=\frac{\alpha_{pn}\left(T_{h}-T_{c}\right)}{R+R_{L}}$$

The electric power output, $P_{out}$, and efficiency, *η*, of a TEG module are given as [80]:

(17) $$P_{out}=q_{h}-q_{c}=-I^{2}R_{L}$$

(18) $$\eta =\frac{P_{out}}{q_{h}}=\frac{I^{2}R_{L}}{\alpha_{pn}IT_{h}+k\left(T_{h}-T_{c}\right)-\frac{1}{2}I^{2}R}$$

**Figure 9:**
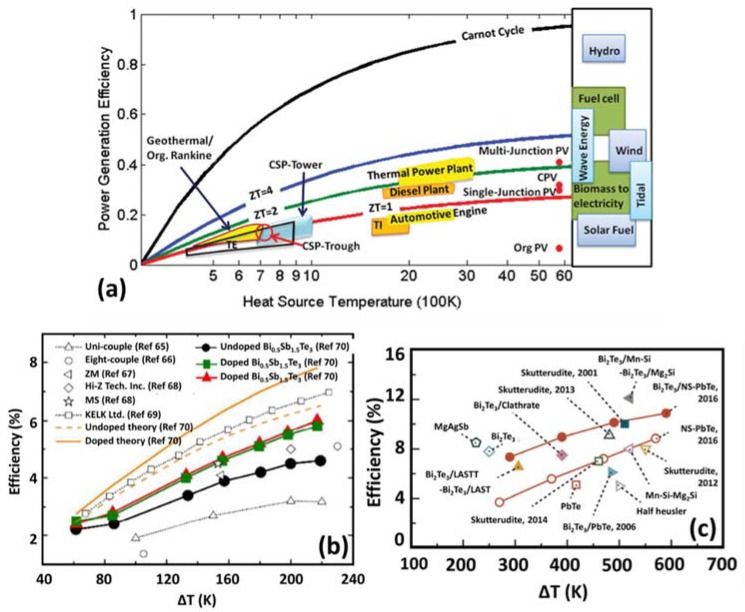
(**a**) Efficiency of thermoelectric generator against other thermal energy conversion technologies. (PV: photovoltaic, CSP: concentrated solar power, Org: Organic, TE: thermoelectric, and TI: thermionic devices). The cold side is considered to be at room temperature, adapted from [82], with permission from © 2012 Royal Society of Chemistry; (**b**) Efficiency reported for some of the state-of-art TEG modules suitable for low temperature thermal energy harvesting application, adapted from [88], with permission from © 2016 Royal Society of Chemistry; (**c**) Efficiency reported for some state-of-art TEG modules suitable for mid to high temperature applications, adapted from [89], with permission from © 2016 Royal Society of Chemistry.

Negative sign in Equation (17) indicates energy released. For maximum value of *η*, *dη*/*d*(*RL*/*R*) should be zero. The maximum value of efficiency, *η_max_*, can be expressed as [81]:

(19) $$\eta_{max}=\frac{T_{h}-T_{c}}{T_{h}}\;\frac{\left[\sqrt{1+\mathrm{ZT}}\right)-1]}{\left[\sqrt{1+\mathrm{ZT}}\right)+\frac{T_{c}}{T_{h}}]}$$

where *T* = ($T_{c}+T_{h}$)/2. The term $\left[\sqrt{1+\mathrm{ZT}}\right)-1]$/$\left[\sqrt{1+\mathrm{ZT}}+T_{c}/T_{h}\right)+]$ represents Joule and other irreversible energy losses and ($T_{h}-T_{c}$)/*T_h_* is Carnot efficiency. Figure 9a compares the efficiency of a thermoelectric generator with other thermal energy conversion technologies [82]. *X*-axis shows hot side temperature on the scale of 100 K. The cold side temperature is considered at room temperature. The graphs also depict the efficiency range of a few other renewable energy technologies in a bar on the right-hand side. Figure 9b shows efficiency reported for some of the state-of-the-art TEG modules suitable for low temperature thermal energy harvesting application [83,84,85,86,87,88]. Figure 9c depicts efficiency reported for some state-of-art TEG modules designed for mid to high temperature range applications [89].

### 2.8. Thermoelectric Generators

**Figure 10:**
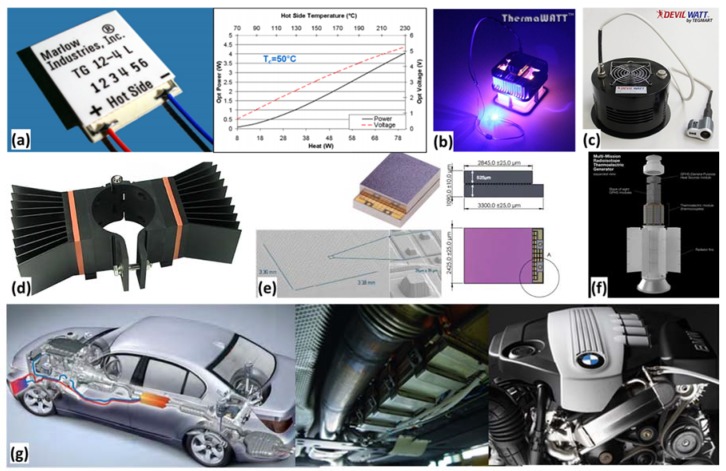
Example commercial thermoelectric generators (TEGs) and their applications. (**a**) TG12-4 TEG from Marlow Industries, Inc. [90]. This TEG of dimension 30 mm × 30 mm generates 4.0 W electrical power across temperature difference of ~180 °C; (**b**) ThermaWatt, a candle powered TEG converts the heat of a candle into electricity [91]. A ThermaWatt of size: 89 mm × 95 mm × 95 mm has power output of 500–800 mW near room temperature; (**c**) DW-DF-10W Camp Stove TEG. This TEG can be placed on top of an open flame source such as a propane stove to charge a battery, power a 12 V DC device, or charge a cell phone [92]; (**d**) EverGen PowerStrap, a product of Marlow Industries, Inc., is used to harvest thermal energy from fluid filled pipes or exhaust stacks for Industrial and Oil/Gas applications [93]; (**e**) MPG-D655 Micropelt thermogenerator chip [94] is a thin film thermoelectric generator having an open circuit voltage of 80 mV/K; (**f**) Radioisotope Thermoelectric Generator (RTG) [22] used as power source for The Mars Science Laboratory rover, Curiosity; (**g**) TEGs to be used on BMW cars for generating electricity from waste heat to improve overall engine efficiency [95].

Thermoelectric generators (TEGs) are the most popular thermal harvesting technology. TEGs are solid-state devices with no moving parts. They produce no toxic discharge and provide reliable operation. The efficiency reported for some of the state-of-the-art TEG modules suitable for thermal energy harvesting application is summarized in Figure 9b [83,84,85,86,87,88]. Figure 10 demonstrates some of the commercial TEGs and their applications.

Figure 10a shows TG12-4 TEG from Marlow Industries, Inc. [90]. Looking at power and efficiency plots, we can notice that this TEG of size 30 mm × 30 mm produces 4.0 W of electrical power across temperature difference of ~180 °C. Figure 10b shows ThermaWatt, a candle powered TEG that converts the heat of a candle to electricity [91]. A ThermaWatt of 89 mm × 95 mm × 95 mm has been reported to have power output of 500–800 mW near room temperature. Figure 10c depicts a DW-DF-10W Camp Stove TEG. This TEG can be placed on top of an open flame source such as a propane stove to charge a battery, power a 12 V DC device, or charge a cell phone [92]. Figure 10d shows EverGen PowerStrap, a product of Marlow Industries, Inc., that is used to harvest thermal energy from fluid filled pipes or exhaust stacks for Industrial and Oil/Gas applications [93]. Figure 10e shows a MPG-D655 Micropelt thermogenerator chip [94]. It is a thin film thermoelectric generator having an open circuit voltage of 80 mV/K. Figure 10f shows a Radioisotope Thermoelectric Generator (RTG) [22] used as a power source for The Mars Science Laboratory rover, Curiosity. RTG provides electrical power for the spacecraft by converting the heat generated from the decay of plutonium-238 (Pu-238) fuel into electricity. Figure 10g shows TEGs to be used on BMW cars for generating electricity from waste heat to improve overall engine efficiency. The vehicle would contain two alternative systems—one unit is being designed for the exhaust system, while the other is intended for the exhaust gas recirculation system [95].

## 3. Pyroelectric Energy Harvesting

The pyroelectric effect may have been predicted more than 2400 years ago, when Greek philosopher Theophrastus described a stone, called lyngourion, having the property of attracting light objects like dust, straws, and bits of wood [96]. The pyroelectric effect originates due to the interaction between polarization and temperature change in some dielectric materials. Certain crystals, such as Tourmaline [97,98] and gallium nitride (GaN) [99,100], are naturally electrically polarized and have non-zero spontaneous polarization at room temperature. Change in temperature of the material causes change in polarization, which is used to generate electricity.

### 3.1. Working Principle

**Figure 11:**
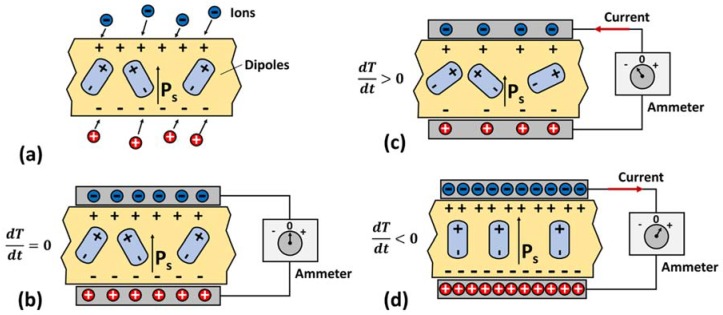
Conceptual model of pyroelectric generator. (**a**) Pyroelectric materials have dipole moments that add up to provide a spontaneous polarization; (**b**) Pyroelectric material between the two conductive electrodes of a capacitor. Temperature is held constant and there is no current in the steady state; (**c**) Increase in temperature decreases spontaneous polarization; (**d**) Decrease in temperature increases spontaneous polarization. Cyclic temperature change in pyroelectric materials generates alternating current. Figure reconstructed from ref. [96].

The concept of pyroelectricity is explained in references [96,101]. As described in Figure 11a, consider a piece of pyroelectric material, such as tourmaline crystal or barium titanate, having crystallographic symmetry axis normal to the flat surfaces. Pyroelectric materials have dipole moments that add up in the direction normal to the flat surfaces to provide spontaneous polarization. The spontaneous polarization (Ps) is defined as net dipole moment per unit volume of the material at room temperature in the absence of an applied electric field. The spontaneous polarization of pyroelectric material allows it to attract nearby particles containing free charges such as electrons or ions. In Figure 11b, the pyroelectric material is kept between the two conductive electrodes of a capacitor. The capacitor charges until the surface charge on the pyroelectric material is neutralized. If the capacitor is now connected to an external electric circuit, it discharges. However, there is no current in the circuit after the system has reached a steady state, provided the temperature of the material is held constant.

For pyroelectric materials, an increase in the temperature of the material causes the net dipole moment, and thus spontaneous polarization to decrease and vice-versa. Consequently, as shown in Figure 11c,d, change in temperature of the material alters the quantity of bound charges. The redistribution of free charges to compensate the change in bound charge results in a current flow, termed as pyroelectric current, in the circuit [96]. By cycling the temperature of pyroelectric materials, therefore, we can generate alternating current.

Comparing Figure 4 and Figure 11, we can note the fundamental difference between thermoelectric and pyroelectric phenomena. Thermoelectricity occurs due to spatial change in temperature; i.e., due to temperature gradient ($\frac{dT}{dx}\neq 0$), whereas pyroelectricity occurs due to a temporal change in temperature; i.e., due to temperature fluctuation ($\frac{dT}{dt}\neq 0$) [102,103].

### 3.2. Pyroelectric Coefficient

The pyroelectric coefficient of a material, under a constant stress and electric field, is defined as [104]:

(20) $$p={\left(\frac{dP_{s}}{dT}\right)}_{\sigma ,E}$$

where σ and *E* denote stress and electric field, respectively, and the letters in subscripts correspond to constant conditions.

Assuming a homogeneous pyroelectric material (constant pyroelectric coefficient) throughout which the temperature *T* at any time is uniform, the electric current generated (Figure 11c,d) from the pyroelectric effect is given as [104,105]:

(21) $$i_{p}=\frac{dQ}{dt}=pA\frac{dT}{dt}$$

where *Q* denotes the pyroelectric charge, $i_{p}$ represents the pyroelectric current, *A* is the surface area of the pyroelectric material, and $\frac{dT}{dt}$ is the rate of temperature change. It should be noted that the current obtained from Equation (21) is under a short circuit condition and the electrodes of the capacitor are positioned as normal to the polar direction. In addition, Equation (21) shows that the pyroelectric current is proportional to the surface area of the material and is independent of its thickness. This happens because the current is simply the rate of change of surface charge, *Q*, and has no relationship with volume of the material.

Using Equation (21), the net charge developed on the electrodes of the capacitor in Figure 11 can be calculated as:

(22) $$Q=\int i_{p}dt=pA\Delta T$$

where Δ*T* is the temperature change. The equivalent capacitance (*C*) of the capacitor is given by [103]:

(23) $$C=\frac{A\varepsilon_{33}^{\sigma }}{h}$$

where $\varepsilon_{33}^{\sigma }$ is the permittivity in the polarization direction at constant stress and *h* is thickness of the material. Using Equations (22) and (23), we can calculate open circuit voltage, *V_OC_*, and total energy, *TE*, stored in a capacitor as:

(24) $$V_{oc}=\frac{Q}{C}=\frac{ph\Delta T}{\varepsilon_{33}^{\sigma }}$$

(25) $$TE=\frac{1}{2}CV_{oc}^{2}=\frac{1}{2}\frac{p^{2}Ah{\left(\Delta T\right)}^{2}}{\varepsilon_{33}^{\sigma }}$$

### 3.3. Figure of Merit

Pyroelectric effect has been used for various applications, such as thermal imaging, laser detectors, radiometers, infrared sensors, gas analysis, fire alarms, and intruder alarms [106,107]. Considering both electrical and thermal properties and depending upon the type of electronics used, three different Figure of merit (*FOM*) have been suggested in the literature [108,109]. For the current sensitive readout, Figure of merit, $FOM_{i}$, is given as [110]:

(26) $$FOM_{i}=\frac{p}{c}=\frac{p}{\rho \;c_{P}}$$

where *c* is the volume specific heat, ρ is density of the material and $c_{P}$ is specific heat. For a voltage sensitive readout, Figure of merit, $FOM_{v}$, is given as [110]:

(27) $$FOM_{v}=\frac{p}{c\;\epsilon_{33}^{\sigma }}$$

where $\epsilon_{33}^{\sigma }$ is dielectric constant (permittivity) of the material in the polarization direction at constant stress. Lastly, for the detectors, where signal to noise ratio is an important parameter, Figure of merit, $FOM_{D}$, is given as [110]:

(28) $$FOM_{D}=\frac{p}{c\sqrt{\epsilon_{33}^{\sigma }tan\delta }}$$

where tan *δ* is the dielectric loss.

### 3.4. Pyroelectric Materials

Pyroelectric materials need to be polar and exhibit spontaneous polarization. Whether a solid material exhibits the pyroelectric effect or not is determined by its crystal structure. Out of thirty two point groups, only ten satisfy following conditions required to exhibit pyroelectricity [96]:

(1) The molecular structure must have a nonzero dipole moment.
(2) The material possesses no center of symmetry.
(3) The material must have either no axis of rotational symmetry or a unique axis of rotational symmetry, not included in an inversion axis.

**Figure 12:**
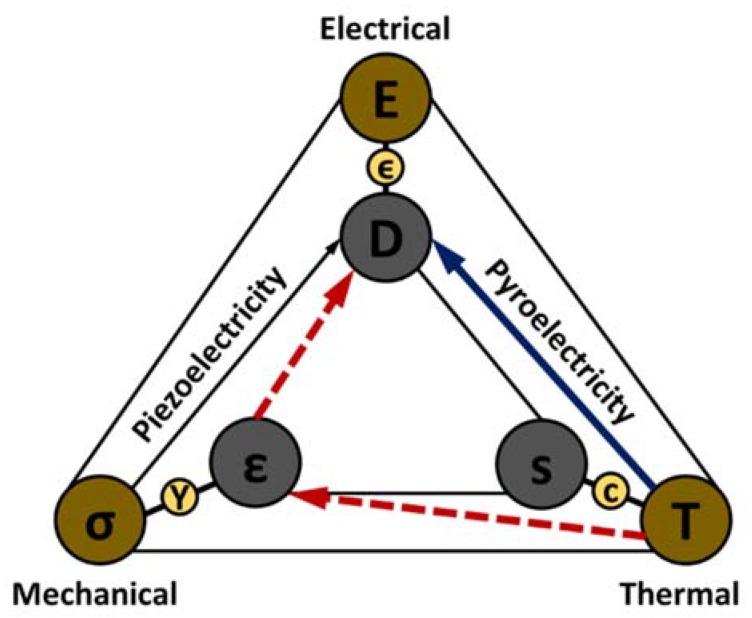
Heckmann diagram illustrating the thermodynamically reversible interactions that may occur among the thermal, mechanical, and electrical properties of a crystal.

Figure 12 depicts a Heckmann diagram illustrating the thermodynamic reversible interactions that may occur among the thermal, mechanical, and electrical properties of a crystal [96,111]. Here *T*, *E*, and *σ* in the outer circles denote temperature, electric field, and elastic stress. A small change in any one of these parameters produces a corresponding change in the others. Variables *s*, *D*, and *ε* in the inner circles denote entropy, dielectric displacement, and strain, respectively, and are an immediate effect of temperature, electric field, and elastic stress defined by their relationship with physical properties heat capacity, *c*, dielectric constant, ϵ, and elastic modulus, *Y*, of the material.

Figure 12 also illustrates two coupled effects: pyroelectricity and piezoelectricity. Pyroelectricity relates temperature with dielectric displacement and piezoelectricity relates stress with dielectric displacement. Note that the pyroelectric effect has two routes: the primary route is shown by a solid blue line and the secondary route is indicated by a dotted red line. The primary pyroelectric effect occurs when material is perfectly clamped and is under constant strain with uniform temperature distribution and no external electric field. This condition is hard to achieve in practice. In most cases, a secondary pyroelectric effect is present, where thermal expansion induces a strain on the material that alters the dielectric displacement via the piezoelectric effect. The total pyroelectric coefficient, consisting of both primary and secondary components, under constant stress condition is given as:

(29) $$p^{\sigma ,E}=p^{\varepsilon ,E}+d_{ij}e_{ij}^{E}\lambda_{i}^{E}$$

where the first term in Equation (29) $p^{\varepsilon ,E}$ shows the primary pyroelectric coefficient under constant strain condition. The second term $d_{ij}e_{ij}^{E}\lambda_{i}^{E}$ represents the secondary pyroelectric coefficient, and tensors $d_{ij}$, $e_{i}$, and $\lambda_{i}$ are the piezoelectric coefficient, elastic constant, and thermal expansion coefficient, respectively.

**Figure 13:**
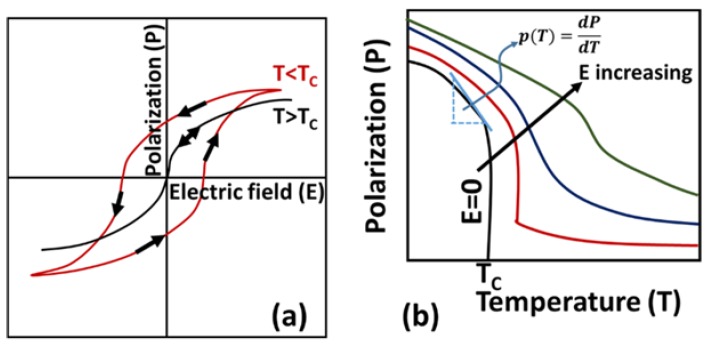
(**a**) Polarization (P) vs. applied electric field (E) responses of a ferroelectric material above and below the Curie temperature, *T_C_*. Hysteretic behavior can be seen at temperature below *T_C_*; (**b**) Variation of polarization with respect to temperature at different applied electric fields. The pyroelectric coefficient (gradient of polarization versus temperature, $\frac{dP}{dT}$) is highest near the Curie temperature. Figures reconstructed from ref. [112].

It is interesting to note that all pyroelectric materials are piezoelectric, but not all piezoelectrics are pyroelectric. Also, all ferroelectric materials are both pyroelectric and piezoelectric [103]. Ferroelectric materials can switch the orientation of its spontaneous polarization when the direction of the applied alternating electric field is reversed. However, reversal is often accompanied with ferroelectric hysteresis (Figure 13a). Most ferroelectric materials exhibit a transition temperature (called Curie temperature, *T_C_*) above which they transform to non-ferroelectric or paraelectric phase. At Curie temperature, the spontaneous polarization of a ferroelectric material ceases to zero. Ferroelectric materials are of special interest in the field of pyroelectrics because the pyroelectric coefficient (gradient of polarization versus temperature, $\frac{dP}{dT}$) is very high near the Curie temperature (as shown in Figure 13b).

### 3.5. Thermodynamic Cycles

**Figure 14:**
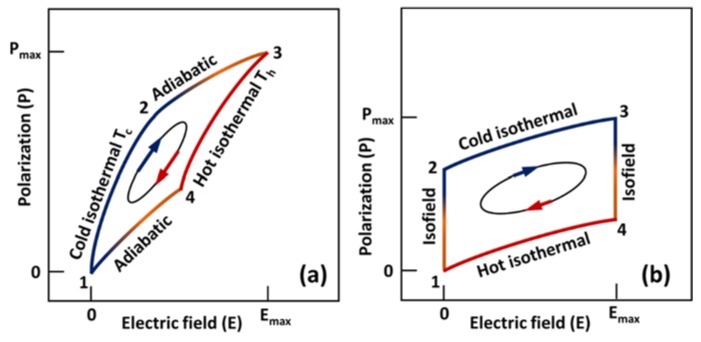
Thermodynamic cycles for pyroelectric generator. (**a**) Carnot cycle; (**b**) Ericsson cycle.

There are a variety of thermodynamic cycles proposed in the literature for pyroelectric generators [113,114,115]. Details can be found in references [116,117]. In this study, we will discuss two of the most important ones: Carnot cycle [118,119] and Ericson cycle [101,120]. These cycles are shown is Figure 14.

#### 3.5.1. Pyroelectric Carnot Cycle

The Carnot cycle is considered an ideal cycle in thermodynamics and it imposes a maximum limit on the efficiency of a heat engine. The pyroelectric Carnot cycle, like the gas Carnot cycle, consists of two isothermal processes and two adiabatic processes. As shown in Figure 14a, path 1–2 and 3–4 represent isothermal processes and path 2–3 and 4–1 represent adiabatic processes. State 1 represents the initial condition of the pyroelectric material under no external electric field and at temperature $T_{c}$. The material is then exposed to an electric field and its strength is increased isothermally (temperature remains constant) from state 1 to state 2 and then adiabatically (no heat interaction with the surroundings) from state 2 to state 3. State 3 represents state of maximum allowable external electric field, which is subjected to the dielectric strength of the material at the operating temperature. Electric field is then decreased isothermally from state 3 to state 4 and then adiabatically until it reduces to zero (state 1). The Carnot cycle efficiency of a heat engine operating between a hot reservoir at temperature *T_h_* and a cold reservoir at temperature $T_{c}$ is given as:

(30) $$\eta_{carnot}=1-\frac{T_{c}}{T_{h}}$$

The Carnot cycle is practically difficult to achieve because of the requirement for adiabatic temperature changes and two isothermal process. The Carnot cycle, however, provides the maximum possible efficiency of a pyroelectric generator and the Carnot efficiency is often used to evaluate relative efficiency of the other thermodynamic cycles.

#### 3.5.2. Pyroelectric Ericsson Cycle

The pyroelectric Ericsson cycle is illustrated in Figure 14b. For pyroelectric energy conversion, the Ericsson cycle was first introduced by Olsen in 1980 and then extensively studied by other researchers in subsequent years [121]. As shown in Figure 14b, this cycle consists of two isothermal processes (2 to 3 and 4 to 1) and two constant electric field processes (1 to 2 and 3 to 4). State 1 represents the initial condition of the pyroelectric material under zero electric field and at temperature *T_h_*. The material is cooled under no electric field condition until temperature $T_{c}$ is achieved (state 2). The electric field is then applied and increased isothermally until the electric field increases to *E_max_* (state 3). This process is followed by isofield heating, where the material is heated from state temperature $T_{c}$ to *T_h_* under a constant electric field. Lastly, the electric field is decreased from *E_max_* to zero, while the temperature of the material is maintained at constant temperature *T_h_*.

The electric work output *W_cycle_* from a pyroelectric cycle can be given as [122]:

(31) $$W_{cycle}=-\left(T_{h}-T_{c}\right)\int_{0}^{E_{max}}pdE$$

Also, heat inflow from the hot reservoir can be obtained as [122]:

(32) $$Q_{in}=c\;\left(T_{h}-T_{c}\right)+\int_{0}^{E_{max}}pT_{h}dE\;$$

Thermodynamic efficiency can then be determined as [122]:

(33) $$\eta =\frac{\left|W_{cycle}\right|}{Q_{in}}$$

Therefore,

(34) $$\eta =\frac{\int_{0}^{E_{max}}pdE}{c+\frac{T_{h}}{\left(T_{h}-T_{c}\right)}\int_{0}^{E_{max}}pdE}$$

#### 3.5.3. Modified Ericsson Cycle or Olsen Cycle

**Figure 15:**
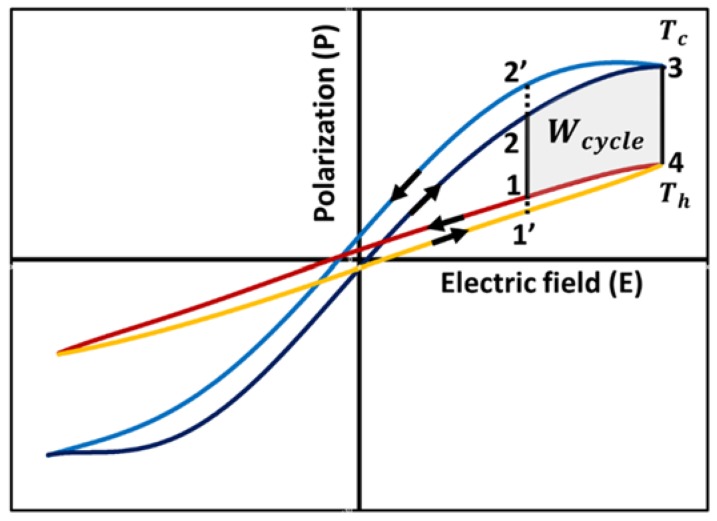
Modified Ericsson Cycle or Olsen cycle for pyroelectric generator.

Ericsson cycle described above does not account for hysteresis losses. As shown in Figure 14, most pyroelectric materials exhibit hysteresis. Figure 15 shows typical polarization-electric field behavior of a pyroelectric material at two different temperatures: $T_{h}$ and $T_{c}$. As indicated in Figure 15, area bounded by cycle 1-2-3-4 is less than that bounded by the ideal Ericsson cycle 1′-2′-3-4. The cycle described by path 1-2-3-4 can be modified as an Ericsson Cycle or an Olsen cycle [121]. Table 2 compares the $W_{cycle}$ based on the Olsen cycle for different pyroelectric materials.

### 3.6. Pyroelectric Generators

**Figure 16:**
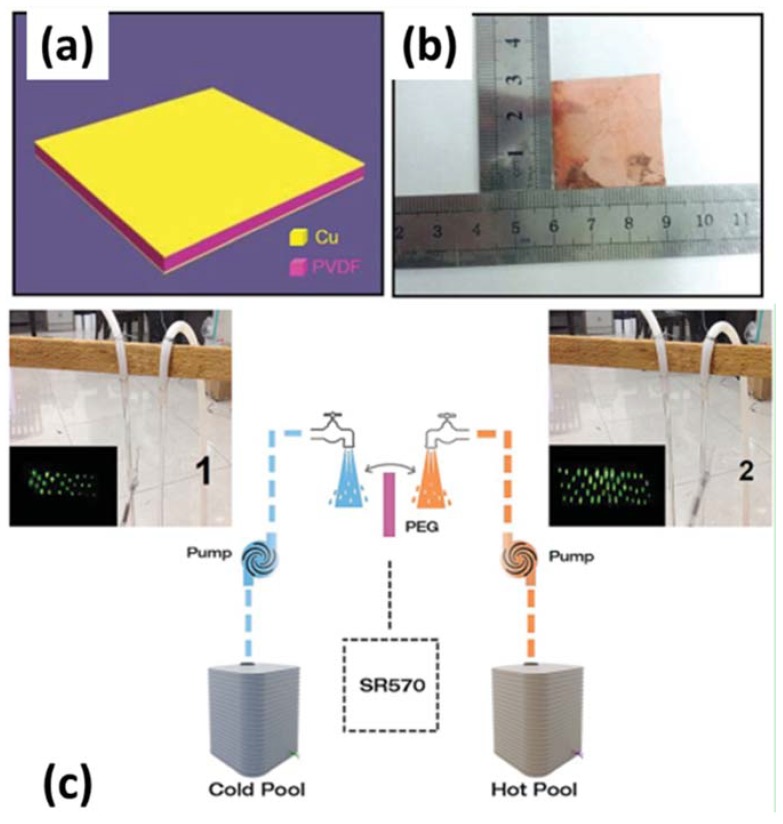
Leng et al.’s pyroelectric generator based on the PVDF film. (**a**) Schematic diagram; (**b**) Digital photograph; (**c**) Experimental setup for testing pyroelectric generators (PEG) under the alternating contact of hot and cold water flow adapted from [138], with permission from © 2014 Royal Society of Chemistry.

Pyroelectric generators (PEGs) require cyclic variation in temperature in order to operate. In most cases, this is achieved by oscillating PEG between hot and cold fluids, as shown in Figure 16c [138]. The heat transfer rate between the device and working fluid often limits the frequency of operation (~1 Hz), making PEGs low power density devices. There are limited experimental studies reported in the literature on PEGs. Olsen and co-workers demonstrated PEG prototypes with a power output of 1 mW and 40 mW having efficiency of around 0.4% [120,129,139]. Olsen et al. [128] also demonstrated a cascaded pyroelectric generator built using different grades of lead zirconate stannate titanate (PZST), providing maximum power output of 33 Watts per liter of pyroelectric materials at 0.26 Hz. The maximum thermodynamic efficiency of 1.05% was obtained at 0.14 Hz, which was 12% of the Carnot efficiency [128]. More recently, in 2010, Nguyen et al. [134] demonstrated experimental studies on a PEG prototype built using co-Polymer 60/40 P(VDF-TrFE). This study reported a maximum power density of 10.7 W/L of pyroelectric material at a frequency of 0.12 Hz with temperature oscillating between 70.5 °C and 85.3 °C.

In order to improve the heat transfer rate and thus to increase the oscillating frequency, a few recent studies have proposed thin film based pyroelectric generators. Yang et al. [140] reported a PZT thin film (175 mm thickness) based pyroelectric generator, exhibiting a pyroelectric coefficient of approximately −800 mC/m^2^·K with a maximum power density of 0.215 mW/cm^3^. Leng et al. [138] reported a PEG based on polyvinylidene fluoride film for harvesting the heat energy from hot/cold water. They reported maximum output open-circuit voltage of 192 V and short-circuit current of 12 mA under temperature change of 80 °C. The highest output power density was found to be 14 μW/cm^2^ or 1.08 W/cm^3^. Figure 16a,b show Leng et al.’s pyroelectric generator based on the PVDF film [138].

Few researchers have attempted to build hybrid thermal harvesters incorporating both pyroelectrics and piezoelectric effects [141]. This is possible because all pyroelectric materials are piezoelectric. One such piezoelectric–pyroelectric hybrid device has been recently investigated by Lee at el. [141]. This nano-generator is based on a micro-patterned piezoelectric P(VDF-TrFE) polymer, micro-patterned PDMS carbon nanotube (CNTs) composite and graphene nanosheets. PDMS-CNT makes the device flexible and stretchable. It also serves as a robust electrode on the base of the device. Graphene used as the top flexible electrode allows fast thermal response due to its high thermal conductivity.

## 4. Thermomagnetic Energy Harvesting

Thermomagnetic energy harvesting relies on the effect of heat on the magnetic properties. Increasing temperature of a magnetocaloric material, such as Gadolinium (Gd), Gd_5_(Si_2_Ge_2_) etc., causes magnetic dipole moments to disorient due to thermal agitation, resulting in a decrease in the magnetization of the material. On the other hand, decreasing the temperature of the material allows magnetic dipoles to reorient and therefore it increases magnetization in the material. The effect is most prominent in ferromagnetic materials, which undergo a phase change near the transition temperature. The transition temperature, also termed as the Curie temperature or Curie point, of ferromagnetic materials refers to the condition where magnetization disappears and material transforms into paramagnetic state. It is important to note that ferromagnetic Curie temperature is different that the ferroelectric Curie temperature that was discussed earlier.

### 4.1. Working Principle

**Figure 17:**
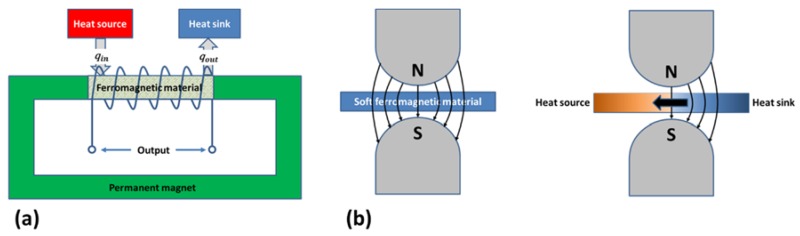
The working mechanism of active (**a**) and passive (**b**) thermomagnetic energy conversion devices. Adapted from [142], with permission from © 2018 Elsevier.

Electricity generation from heat via thermomagnetic effect can be accomplished in two different ways. The first method is direct energy conversion, where thermal energy is directly converted into electrical energy. Such systems are referred to as active thermomagnetic devices or thermomagnetic generator. The second conversion method is via an intermediate mechanical stage and such systems are called as passive thermomagnetic devices or thermomagnetic motor. Figure 17 shows the working mechanism of the active and passive thermomagnetic energy conversion devices [142].

As shown in Figure 17a, an active thermomagnetic system consists of a C-shaped permanent magnet or an electromagnet, a ferromagnetic material placed as a shunt between the poles of the permanent magnet, and a winding around of the shunt material. The shunt element is heated and cooled, alternately, across the Curie temperature of the material. Rise in shunt temperature reduces the magnetization and the magnetic flux thereof. Decrease in temperature produces the reverse effect; i.e., the magnetization and the magnetic flux increases. The cyclic heating and cooling of the shunt material results in continuously changing magnetic flux, which induces a voltage across the two ends of winding per Faraday’s law of electromagnetic induction. When winding is connected to an external resistor, electrical power is produced.

The passive thermomagnetic devices, called a thermomagnetic motor or Curie motor, convert thermal energy into mechanical energy in the form of rotary or linear motion. Therefore, in order to produce electricity, an electromechanical generator is needed. As shown in Figure 17b, a thermomagnetic motor, in the simplest form, consists of a magnetic circuit with a movable armature made-up of soft ferromagnetic material. When the entire armature is at the same temperature, the magnetic permeability is uniform and the magnetic forces from the opposite directions are balanced. If a portion of the armature is now heated above the Curie temperature using a heat source, while maintaining the other portion below the Curie point using a heat sink, it causes a permeability difference between hot and cold spots. This exerts a net unbalanced force on the armature, which results in linear or rotary motion. A restoring mechanism, such as mechanical spring, is needed to restore the initial condition.

### 4.2. Thermomagnetic Cycle

**Figure 18:**
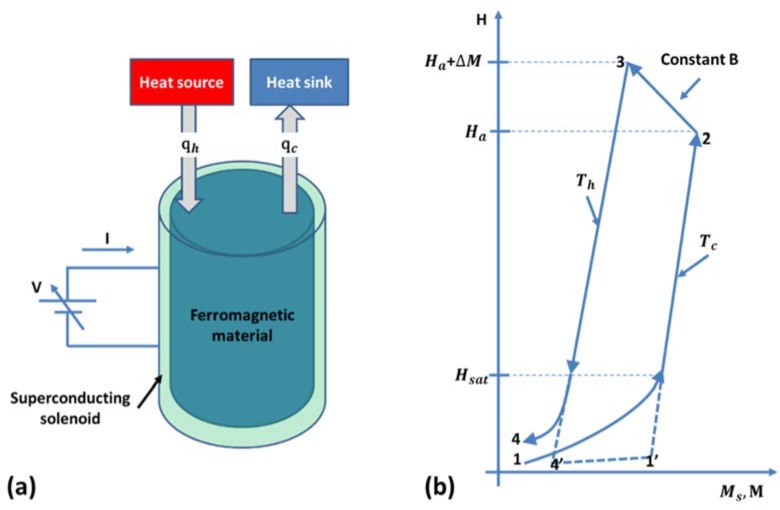
(**a**) A conceptual model of Solomon’s thermomagnetic generator. (**b**) Thermomagnetic cycle. Adapted from [142], with permission from © 2018 Elsevier.

For the thermomagnetic generator described in Figure 17, the external magnetic field is fixed throughout the thermodynamic cycle. Solomon [143] recommends a thermomagnetic cycle described in Figure 18b, where the applied magnetic field is also cycled as the material is thermally cycled. In Figure 18a, a ferromagnetic material is located inside the magnetic field created by a superconducting solenoid. A variable voltage source is used to produce voltage v and current i in the windings around the solenoid. The generated magnetic fields *B* and *H* are given [142,143] by:

(35) $$v=nA\frac{dB}{dt}$$

(36) $$i=\frac{HL}{n}$$

where n is number of windings of the solenoid, A is its cross-sectional area, $\frac{dB}{dt}$ is rate of change of magnetic induction in the ferromagnetic material, *R* is resistance of wire, and L is length of solenoid.

The magnetic induction B in the ferromagnetic material is related to magnetization and applied magnetic field as [142,144]:

(37) $$B\left(H,T\right)=\mu_{0}\left[H+M\left(H,T\right)\right]$$

where $\mu_{0}$ is permeability of free space, H is applied magnetic field, and M is magnetization, which is function of applied field and its temperature. For a thermomagnetic process, the power P and energy E supplied by voltage source to the unit volume of ferromagnetic material can be calculated as [142,143,145]:

(38) $$P=\frac{vi}{AL}=H\frac{dB}{dt}$$

(39) $$E=\int Pdt=\int H\;dB=\mu_{0}\int H\;dH+\mu_{0}\int H\;dM$$

Since for a cyclic process ∮H dH=0, the net work done, $w_{cycle}$ by the voltage source during the thermomagnetic cycle is given as:

(40) $$w_{cycle}=\mu_{0}\oint H\;dM$$

Assuming a net gain of energy by the voltage source, work output $w_{out}$ produced by the thermomagnetic generator would be negative and is given by:

(41) $$w_{out}=-\mu_{0}\oint H\;dM$$

The efficiency of the thermomagnetic generator is given as:

(42) $$\eta_{max}=\frac{w_{out}}{q_{in}}=\frac{\mu_{0}\oint H\;dM}{q_{in}}$$

where $q_{in}$ is heat inflow during the heating portion of the thermomagnetic cycle.

From the first law of thermodynamics, the heat input to a system is equal to the sum of increase in internal energy and the work done by system. For a ferromagnetic material, the change in internal energy is associated with two effects: (i) change in temperature and (ii) change in magnetic entropy. Therefore, we can write [146]:

(43) $$q_{in}=\rho \int_{T_{c}}^{T_{h}}C_{p}\left(T\right)\;dT+T\int dS_{m}$$

where $C_{p}$ is the specific heat of the material and $S_{m}$ is the magnetic entropy. When the applied magnetic field *H* is small, the first term $\rho \int_{T_{c}}^{T_{h}}c_{p}\left(T\right)dT$ is much larger than $T\int dS_{m}$, and therefore later can be ignored [147,148]. Efficiency (or absolute efficiency) of the thermomagnetic cycle is expressed as:

(44) $$\eta \;\left(or\;\eta_{abs}\right)≅\frac{\mu_{0}\oint H\;dM}{\rho \int_{T_{c}}^{T_{h}}C_{p}\left(T\right)\;dT}$$

The relative efficiency with respect to Carnot efficiency of a thermomagnetic cycle can also be calculated as:

(45) $$\eta_{rel}=\frac{\eta_{abs}}{\eta_{carnot}}=\frac{\eta_{abs}}{1-\frac{T_{c}}{T_{h}}}$$

Elliott [149] derived the maximum power output of a thermomagnetic generator in terms of magnetic characteristics of the permanent magnet as:

(46) $$P_{max}=\frac{1}{8}\omega V\mu_{r}H^{2}$$

where *V* and $\mu_{r}$ denote volume and permeability of permanent magnet, and it is assumed that the temperature of shunt varies in a sinusoidal manner at an angular frequency ω. Elliott [149] also suggested that the maximum theoretical efficiency of a thermomagnetic generator operating between hot-side temperature close to the Curie temperature ($T_{h}\approx T_{C}$) and cold-side temperature $T_{c}=T_{C}-∆T$ can be expressed as:

(47) $$\;\eta_{max}=\frac{\pi }{4\sqrt{2}}\frac{∆T}{T_{C}}=0.55\times \mathrm{Carnot}\;\mathrm{efficiency}$$

### 4.3. Thermomagnetic Materials

The thermomagnetic effect in a ferromagnetic material is highest near its Curie temperature. At Curie temperature, ferromagnetic materials undergo phase transition from magnetic to non-magnetic states. The phase transition is usually classified as first order or second order. The second-order phase transition is gradual and occurs without the coexistence of multiple phases. On the other hand, the first order phase transition involves the occurrence of two phases in equilibrium in the transition zone. When a material with first-order transition is heated, it exhibits an abrupt phase change that transforms it from a strongly magnetic to a weakly magnetic phase. The first order transition materials normally exhibit higher thermomagnetic effect than the second order transition materials; however, they are normally accompanied with thermal and magnetic hysteresis, which reduces the overall work output.

Gadolinium (Gd) is by far the most recommended thermomagnetic material [143,146,150] for thermal energy harvesting for two principal reasons. First, Gd has a Curie temperature of ~293 K [151]; therefore, it can be utilized near room temperature. Second, Gd undergoes second order phase transition from the ferromagnetic and paramagnetic state and has no thermal or magnetic hysteresis. However, since Gd is paramagnetic at room temperature, it normally requires a heat sink (or a refrigerator). Generally, heat sources are more conveniently available (for example, in the form of waste heat) than the heat sink; therefore, thermomagnetic materials having Curie temperature slightly higher than room temperature are desired. Srivastava et al. [152] have recently recommended the multiferroic Heusler alloy, Ni_45_Co_5_Mn_40_Sn_10_ as an alternative to Gd. The Heusler alloy undergoes first order transition from a strongly ferromagnetic austenite phase to a weakly ferromagnetic martensite phase at critical temperature of 408 K [153,154]. Though the work output per thermomagnetic cycle is higher for a Heusler alloy than gadolinium, both the materials have been found to exhibit similar thermomagnetic efficiency [148]. This happens because the thermal energy requirement at critical temperature for first order phase transformation is dominated by latent heat, whereas the second order phase transition is mainly governed by sensible heat. Heusler alloy, therefore, needs larger heat input per thermomagnetic cycle than gadolinium.

Few other thermomagnetic materials have been reported in the literature such as Gd_5_Si_2_Ge_2_ [155], GdFe_6_Al_6_ [156] and Gd based other binary and ternary compounds [157,158,159], MnAs and its related compounds [160,161,162], and lanthanide transition-metal-based compounds [163,164,165,166,167,168]. Most of these materials exhibit first-order transition and they have been reported to demonstrate a significant magnitude of thermomagnetic effect. However, their performance in thermomagnetic energy harvesters has not been yet thoroughly examined. In addition to the thermomagnetic effect, several other considerations need to be taken in to account when a material is used in a thermal harvester. For example, materials having large thermal and magnetic hysteresis are not recommended. The materials having small specific heat and large thermal conductivity are preferred, as it ensures rapid heat exchange and temperature change.

### 4.4. Thermomagnetic Devices

**Figure 19:**
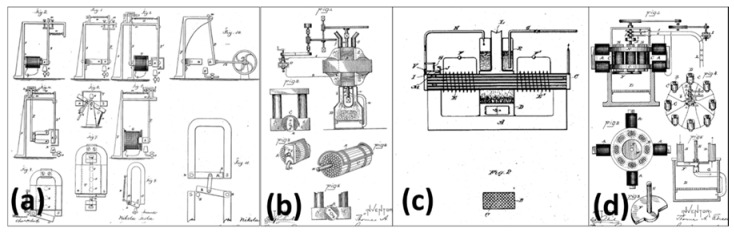
Few patents [169,170,171,172] issued in late 19th century describe concepts for converting heat into mechanical or electrical energy using thermomagnetic effect. (**a**) Thermomagnetic motor by Nikole Tesla (US Patent 396121); (**b**) Pyromagnetic motor by Thomas Edison (US Patent 380100); (**c**) Pyromagnetic electric generator by Nikole Tesla (US Patent 428057); (**d**) Pyromagnetic generator by Thomas Edison (US Patent 476983). Adapted from [142], with permission from © 2018 Elsevier.

The fact that heat alters magnetic properties of ferromagnetic materials is known for a long time. Figure 19 depicts few patents [169,170,171,172] issued in the late 19th century describing concepts for converting heat into mechanical or electrical energy using the thermomagnetic effect. As it can be seen, most of these devices had very complex designs and they used iron as the working ferromagnetic material. Iron has very high Curie temperature, around 1000 K. Achieving such a high temperature requires lot of fuel and high temperature may cause thermal degradation of the permanent magnets used in the device. In addition, since the thermodynamic efficiency of heat engine is proportional to $\frac{∆T}{T}$, where T denotes the working temperature, as transition temperature increases the efficiency of the thermomagnetic device decreases, which makes the convention ferromagnetic materials such as iron, cobalt, and nickel unsuitable for thermomagnetic devices [142].

**Figure 20:**
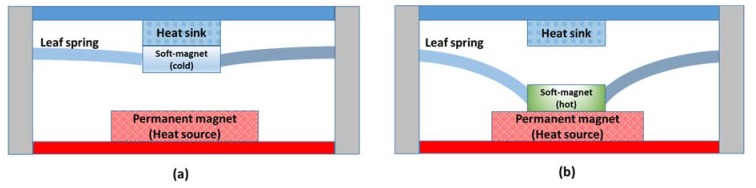
Thermomechanical actuator reported by Ujihara et al. (**a**) Soft magnet is ferromagnetic; (**b**) soft magnet is paramagnetic, adapted from [142,179], with permission from © 2018 Elsevier.

**Figure 21:**
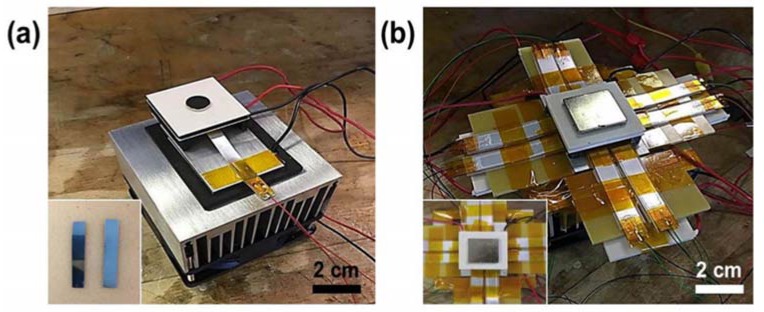
Chun et al.’s thermo-magneto-electric generator (TMEG). (**a**) TMEG with a single bimorph cantilever and (**b**) TMEG with arrays of bimorph cantilevers. Figure taken from [180].

Active thermomagnetic devices are not much studied in the literature, possibly because of their requirement of cyclic variation in temperature. Passive thermomagnetic energy harvesters, however, can be made to operate with heat sources at constant temperature. Some of the thermomagnetic motors reported in the literature are Van Der Mass and Purvis’s Curie point motor [173], Murakami and Nemoto’s rotary thermomagnetic motor [174], Takahashi’s thermomagnetic engine [175,176], and Palmy’s floating thermomagnetic wheel [177,178]. Figure 20 shows the conceptual model of a thermomechanical actuator presented by Ujihara et al. [179]. The device consists of a small gadolinium piece suspended on a leaf spring near a relatively large permanent neodymium magnet. As shown in Figure 20, the permanent magnet is in contact with a heat source, whereas gadolinium is initially in contact with a heat sink. When gadolinium is below its Curie temperature, it is magnetic and is attracted by the permanent magnet. After it contacts the heat source, its temperature rises above its Curie temperature and it becomes paramagnetic. Gd is then pulled away by the leaf spring, bringing it back in contact with the heat sink. Eventually, its temperature goes below the Curie temperature and it starts to regain its magnetism. When the magnetic force is larger than the spring force, the permanent magnet pulls Gd towards the heat source and the cycle continues. A similar thermomagnetic device has been recently reported by Chun et al. for thermal energy harvesting and for enhancing the cooling rate of solar panels employed in unmanned aerial vehicles. The device shown in Figure 21a is composed of a single bimorph cantilever and was found to enhance the cooling rate of a cooling object by 1 °C per min against the normal dissipation. The device shown in Figure 21b consists of arrays of bimorph cantilevers and it was found to produce 158μW output power at a temperature difference of 80 °C.

## 5. Thermoelastic Energy Harvesting

The concept of thermal strain is well known. Heating or cooling a material normally causes dimensional change, which can be used to produce work. A special class of materials, called shape memory alloys (SMAs), produce substantial stress on heating and therefore they are most suited for such application. At a certain critical temperature, SMAs such as NiTi and Cu-Zn-Al, undergo martensitic transformation, which is a displacive transformation dominated by shear distortions of the crystal lattice [181]. Transformation occurs from the martensite phase (stable at low temperature) to the austenite phase (stable at high temperature), which has drastically different mechanical properties, leading to a large deformation, up to 10%, in the working element.

### 5.1. Working Principle

**Figure 22:**
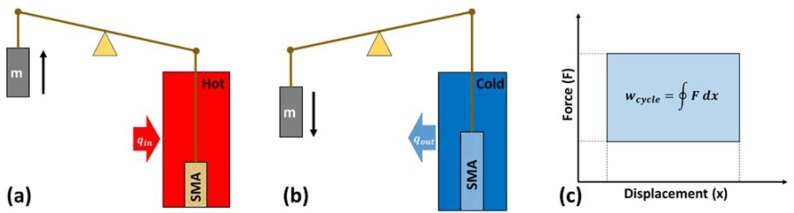
A simplistic conceptual model of a thermoelastic heat engine (**a**) heat absorption process (**b**) heat rejection process (**c**) force-displacement cycle.

Figure 22a,b shows a simplistic conceptual model of a thermoelastic heat engine. The engine contains a shape memory alloy (SMA) as the working element, undergoing contraction and elongation during heating and cooling process, respectively. As SMA element contracts during heating, it applies a force on the attached mass. Force applied by SMA element during contraction substantially exceeds the force applied on the SMA element during elongation, resulting in a network output per cycle as shown in Figure 22c.

### 5.2. Thermoelastic Cycle

**Figure 23:**
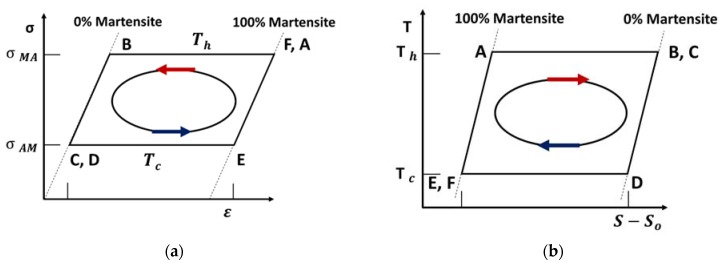
Thermoelastic cycles. (**a**) Stress-strain (*σ*-*ε*) diagram; (**b**) Temperature-entropy (*T*-*S*) diagram.

There are a variety of thermoelastic cycles proposed in the literature for shape memory based heat engines [182,183,184,185,186]. In this study, we will discuss only the Stirling cycle, which is shown in Figure 23. Figure 23a shows a stress-strain (*σ*-*ε*) diagram and Figure 23b shows a temperature-entropy (*T*-*S*) diagram of the thermoelastic cycle. Thermoelastic Stirling cycle consists of two iso-stress heat transfer processes and two isothermal phase change processes. Thermal expansion effects will be disregarded in this cycle and all the processes will be considered thermodynamically reversible.

Process A–B: Process A–B represents martensitic to austenitic phase transformation. State A denotes the condition where the SMA element has just reached the critical temperature ($T_{h}\approx T_{C}$) and it is completely in Martensitic phase. As the process continues, phase transformation occurs, such that temperature and stress remain constant at $T_{h}$ and *σ*_MA_, respectively.

Process B–C: The SMA element is relieved from the stress at level *σ*_MA_ to level *σ*_AM_ at the constant temperature $T_{h}$.

Process C–D: State C represents the condition where martensitic to austenitic phase transformation has completed and the SMA element is in 100% austenitic phase. As the process continues, the materials are cooled from temperature $T_{h}$ to temperature $T_{c}$, at the constant stress of *σ*_AM_.

Process D–E: The SMA element recovers its original shape undergoing the reverse transformation (austenite to martensite), at constant temperature $T_{c}$.

Process E–F: The SMA element is tensioned from the stress at level *σ*_AM_ to level *σ*_MA_ at the constant temperature $T_{c}$.

Process F–A: The SMA element is heated from temperature $T_{c}$ to $T_{h}$ at the constant stress of *σ*_MA_.

### 5.3. Thermoelastic Efficiency

**Figure 24:**
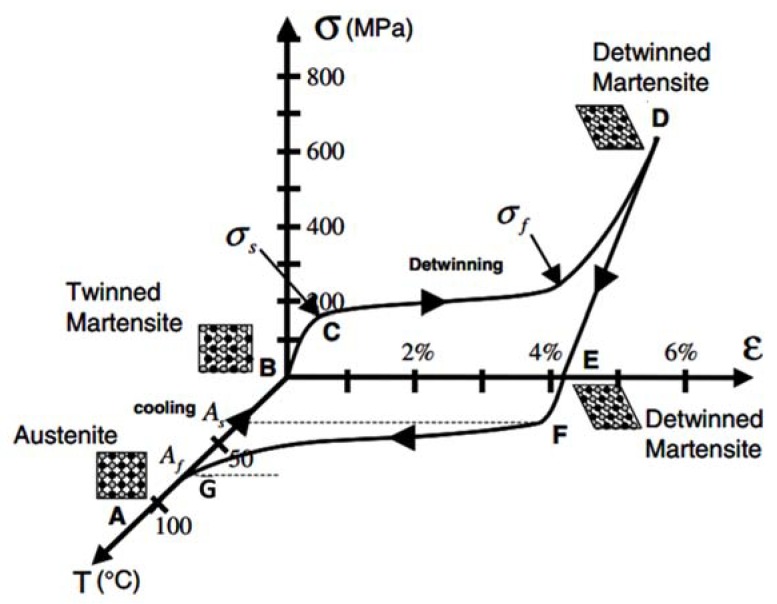
The experimental data in stress-strain-temperature space for a typical NiTi specimen tested under uniaxial loading. Adapted from [188], with permission from © 2018 Springer Nature

With the assumption that all that processes in the cycle A-B-C-D-E-F-A shown in Figure 24 are reversible, we can write heat interaction q during each process as:

(48) $$q_{AB}=T_{h}∆s$$

(49) $$q_{BC}=0$$

(50) $$q_{CD}=-C_{p}\left(T_{h}-T_{c}\right)$$

(51) $$q_{DE}=-T_{c}∆s$$

(52) $$q_{EF}=0$$

(53) $$q_{EF}=0$$

where ∆s denotes change in entropy during a process and $C_{p}$ is specific heat capacity of SMA material.

Heat in and heat out during the complete cycle is given as:

(54) $$q_{in}=q_{AB}+q_{EF}+q_{FA}=T_{h}∆s+C_{p}\left(T_{h}-T_{c}\right)$$

(55) $$q_{out}=q_{BC}+q_{CD}+q_{DE}=T_{c}∆s+C_{p}\left(T_{h}-T_{c}\right)$$

The total work output of the thermoelastic cycle can be calculated using expression:

(56) $$w_{cycle}=q_{in}-q_{out}=\left(T_{h}-T_{c}\right)∆s$$

Thermoelastic energy conversion efficiency is given as:

(57) $$\eta =\frac{w_{cycle}}{q_{in}}=\frac{\left(T_{h}-T_{c}\right)∆s}{T_{h}∆s+C_{p}\left(T_{h}-T_{c}\right)}$$

The thermoelastic cycle described in Figure 24 considers an ideal SMA material that undergoes reversible processes of phase transformations. Energy losses due to any other type of irreversibility were also ignored. In practice, however, the martensitic-austenitic transformations in shape memory materials are not reversible. There are energy losses due to hysteresis during the crystal structure transformations and due to friction in the transmission and driving systems. Muller-Xu-Fedelich-Zanzotto model described in reference [186] accounts for some of the irreversibility and provides a modified thermoelastic energy conversion efficiency equation as:

(58) $$\eta =\frac{\left(T_{h}-T_{c}\right)∆s-2A}{T_{h}∆s+C_{p}\left(T_{h}-T_{c}\right)-A}$$

where *A* is a material constant directly related to stress-strain curve hysteresis; i.e., irreversibility during the phase change process in SMA material [187].

Relative efficiency can now be calculated using:

(59) $$\eta_{rel}=\frac{\eta }{\eta_{Carnot}}$$

Table 3 provides thermoelastic energy conversion efficiency for two popular SMAs: TiNi and CuZnAl, calculated using Equations (58) and (59). It can be noted that, theoretically, the thermoelastic efficiency of SMA engines approaches the Carnot efficiency.

### 5.4. Thermoelastic Materials

The martensitic phase in steel was discovered by Adolf Martens in the 1890s and it is considered a major step towards the discovery of shape memory alloys [188]. In 1949, Kurdjumov and Khandros [189] introduced the concept of thermoelastic martensitic transformation based on the experimental studies on CuZn and CuAl alloys. Later, thermoelastic martensitic transformation was also observed in InTl, CuZn, and NiTi. Out of all these compositions, NiTi (commonly known as Nitinol) became the most popular system because it not only possess shape recovery capability but also has good mechanical properties comparable to common engineering metals [190]. Nitinol is typically composed of ~50–51% nickel (atomic %) and by adjusting the atomic ratio, its transformation temperature can be controlled. The transformation temperature $A_{f}$ (austenite finish) of binary NiTi alloys typically lies in the range of 0 to 100 °C and exhibits a temperature hysteresis of 25–40 °C [191,192]. It has been reported that alloying NiTi with Cu lowers the stress hysteresis without much altering the transformation temperature [193]. NiTiCu alloys are also found to have better fatigue life than NiTi, which makes it suitable for a wide variety of engineering applications [194]. Some SMAs, such as TiPd, TiPt, and TiAu, have been reported to have transformation temperature greater than 100 °C [195]. Commercially available systems include some iron-based SMAs, such as Fe-Mn-Si, and copper-based SMAs, such as Cu-Zn-Al and Cu-Al-Ni.

Thermoelastic effect in shape memory alloys occurs due to their reversible phase transformation behavior at certain critical temperatures. SMAs have two phases: a high temperature phase called austenite (A) and a low temperature phase called martensite (M). These phases have different crystal structures and therefore drastically different mechanical, thermal, electrical, optical, and acoustical material properties [196]. Austenite has generally cubic crystal structure, whereas martensite can be tetragonal, orthorhombic, or monoclinic. The martensitic phase transformation from one structure to the other occurs by shear lattice distortion, forming an assembly of martensitic variants of two kinds: twinned martensite and detwinned martensite [188]. When an SMA is cooled in absence of an applied load, it undergoes a forward transformation, where crystal structure changes from austenite to twinned martensite without a macroscopic shape change. However, if a mechanical load is applied to the material at low temperature, twinned martensitic phase can be detwinned by reorienting a certain number of variants. This results in a macroscopic shape change and the deformation is retained even after the load is released. Heating the material above a certain temperature results in a reverse phase transformation from detwinned martensite to austenite. This leads to complete shape recovery and this phenomenon is known as Shape Memory Effect (SME).

The temperature where forward transformation starts is normally denoted by $M_{s}$ (martensite starts) and the temperature where it finishes is denoted as $M_{f}$ (martensite finishes). Likewise, start and finish temperatures during reverse phase transformation are denoted by $A_{s}$ (austenite starts) and $A_{f}$ (austenite finishes). In addition, the minimum stress in the material (due to an applied load) needed to start the detwinning process is termed as detwinning start stress ($\sigma_{s}$) and sufficiently high stress that results in a complete detwinning of martensite is called detwinning finish stress ($\sigma_{f}$).

Figure 24 depicts the experimental data in stress-strain-temperature space for a typical NiTi specimen tested under uniaxial loading [188]. Looking in stress-temperature (σ−T) plane in Figure 24, point A represents the parent phase where the forward transformation starts ($M_{s}$). The stress-free cooling of austenite below the forward transformation temperatures ($M_{f}$) results in the formation of twinned martensite, denoted by point B. As shown in stress-strain plane in Figure 24, the twinned martensite is now subjected to an applied stress that exceeds the start stress level ($\sigma_{s}$). This initiates the detwinning process (point C), which completes at a stress level $\sigma_{f}$. The material is then elastically unloaded from D to E, but the detwinned martensitic state is retained. Once the stress level reaches zero, the material is heated, which initiates the reverse transformation after the temperature reaches $A_{s}$ (point F). The reverse transformation is completed at temperature $A_{f}$ (point G).

### 5.5. Thermoelastic Devices (SMA Heat Engines)

**Figure 25:**
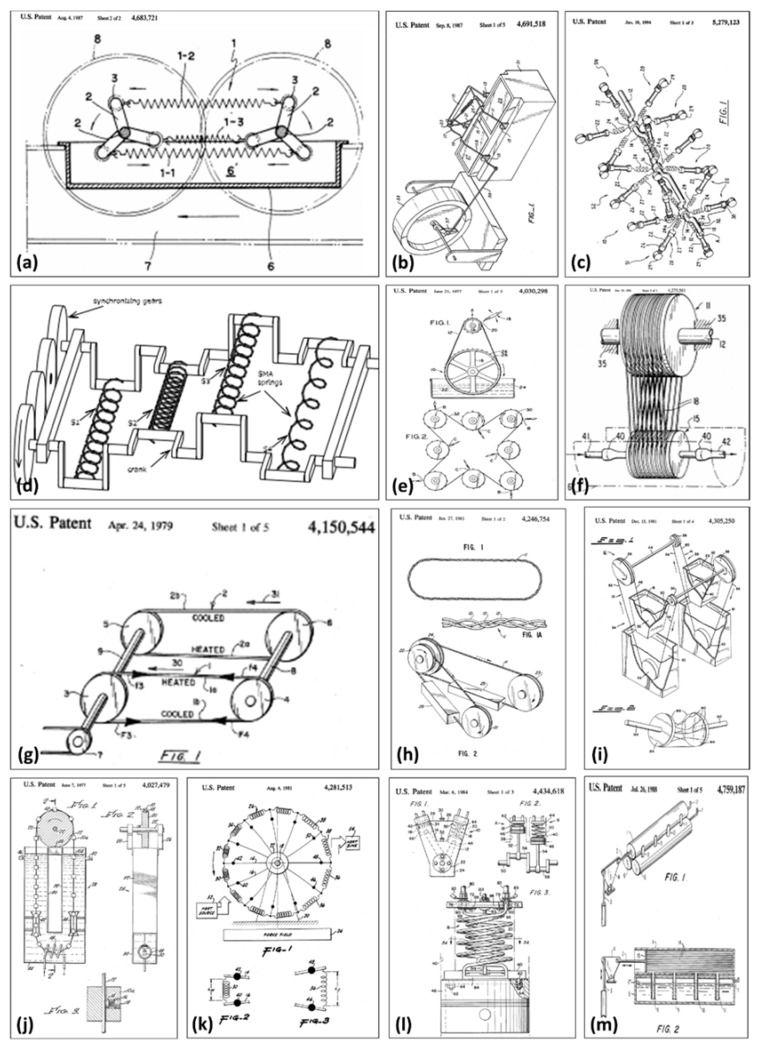
Some early SMA based heat engines (**a**) US Patent 4,683,721 [199]; (**b**) US Patent 4,691,518 [200]; (**c**) US Patent 5,279,123 [201]; (**d**) Twin-crank heat engine [202]; (**e**) US Patent 4,010,612 [203]; (**f**) US Patent 4,275,561 [204]; (**g**) US Patent 4,150,544 [205]; (**h**) US Patent 4,246,754 [206]; (**i**) US Patent 4,305,250 [207]; (**j**) US Patent 4,027,479 [208]; (**k**) US Patent 4,281,513 [209]; (**l**) US Patent 443,461 [210]; (**m**) US Patent 4,759,187 [211].

Thermoelastic based energy conversion devices became popular in the 1970–1980s. More than 100 patents were filed during this period. Some of them are shown in Figure 25. Historically SMA heat engines have been divided into four categories: offset crank engines, turbine engines, field engines, and miscellaneous engines [197,198]. Figure 25a–d show some SMA based crank engines [197,199,200,201,202]. They contain SMA actuators connected eccentrically to the output shaft. This mechanism converts a reciprocating linear motion of an SMA actuator into continuous rotary motion. SMA actuators are trained to form extension springs. An electric generator can be used to produce electricity from a mechanical rotation. Turbine or pulley engines use continuous belts of SMA wire and metal pulleys as the driving mechanism. A portion of SMA wire in one pulley is heated, which produces tension in the wire, causing the pulley to rotate. Figure 25e–i show some SMA based pulley engines [203,204,205,206,207]. SMA based field engines work against a recovering force, such as a gravitational or magnetic field. They normally contain a series of active elements that interact with the force field, weights for the gravitational field and magnets for the magnetic field. These active elements are connected along a loop of SMA wire. A section of the loop is heated that creates contraction and increases the element density in that area. The denser section is affected more by the force field, leading to an unbalanced force causing a rotation. Figure 25j,k show some SMA based field engines [208,209]. Figure 25l,m show some SMA based reciprocating engines [210,211]. They have a relatively simpler design as linear motion is produced by cyclic heating of SMA elements. However, these devices encounter deployment changes as heating and cooling fluids need to be circulated over the SMA elements to produce thermal cycling.

Early devices did not gain much popularity possibly because of the bulky design and low energy conversion efficiency. Numerous theoretical studies [212,213,214,215] investigated the technical feasibility of thermoelastic devices in regards to their work output and efficiency. Thermal to mechanical energy conversion efficiency between 2% and 9% was predicted using a variety of theoretical models based on thermal transport and mechanical measurements [216].

**Figure 26:**
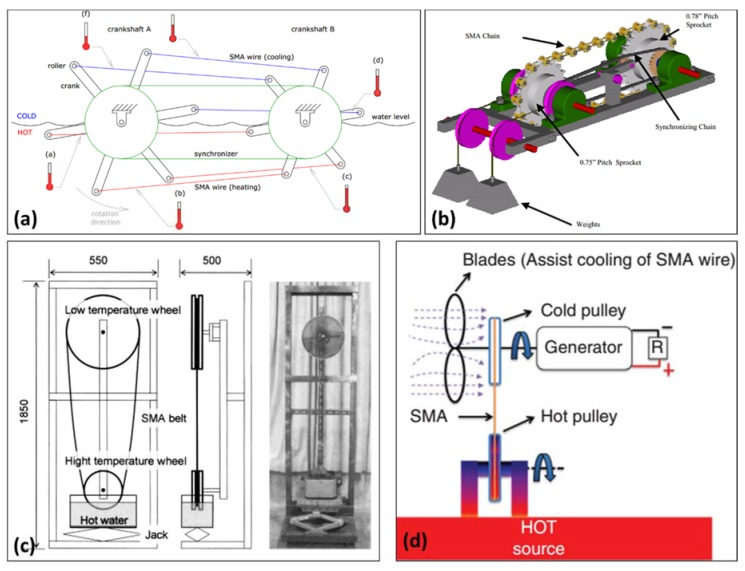
(**a**) Schiller’s SMA engine [217]; (**b**) Wakjira’s SMA engine with driving roller-chain [218]; (**c**) Sato et al.’s large scale SMA heat engine, adapted from [220], with permission from © 2018 John Wiley; (**d**) Avirovik et al.’s miniature SMA engine [221].

Thermoelastic based energy harvesting technique remained dormant for quite some time in the late 20th century. Low interest in this technology might be the result of scalability concerns related to SMA devices. Small prototypes developed in the research laboratories showed promising results, but the scaled-up versions did not produce significant power to justify their cost of deployment. In 2001, researchers at Virginia Tech, USA attempted to revive this technology and proposed a refined engine design [217] along with a dedicated driving mechanism [218] to reduce frictional losses and slip. Figure 26a shows SMA heat engine proposed by Schiller [217]. Schiller’s SMA engine consists of two parallel crankshafts, each supporting multiple evenly spaced cranks. Cranks of the first shaft are aligned with like oriented but shorter cranks on the second shaft, as shown in Figure 26a. A synchronizer, in the form of a timing belt and two identical pulleys, is used to prevent the relative rotation between the two shafts. SMA wires are stretched between rollers located at the end of each crank. During operation, half of the engine’s wires are submerged in the hot region while the other half remains cold. Wires on the hot side contract, whereas wires on the cold side elongate. Since the force generated due to heat recovery exceeds the force required for cooled deformation, the recovery process generates strain in the cold wires, making the engine propels. Figure 26b shows another SMA heat engine design proposed by Wakjira [218]. Wakjira’s engine consists of a SMA driving roller-chain, two driven plastic sprockets whose teeth engage the rollers of a SMA chain, a synchronizer chain, and other components as shown in Figure 26b. More information about the design and operation can be found in ref. [218].

Some research has attempted to develop a thermo-mechanical actuator based on SMAs [217,218]. These systems were designed to convert thermal energy into mechanical energy in the form of linear or rotary motion. Later, few attempts were made to build hybrid thermal engines where SMA systems were integrated with a Piezoelectric system [219]. In most of these devices, SMA elements consumes heat to produce mechanical work, which is eventually converted into electrical energy using some electromechanical converters like an electric generator or a piezoelectric bimorph. More recently, Sato et al. [220] developed a pulley based SMA heat engine shown in Figure 26c. The SMA engine consisting one SMA belt (width: 5 mm, thickness: 0.7 mm, length: 2.3 m) was found to produce a maximum power output of 300 mW. The maximum power output increased to 1.16 W with five parallel SMA belts [220]. Avirovik et al. [221] presented an experimental study on a miniature SMA engine (Figure 26d). The SMA engine was reported to operate near room temperature with hot-side temperature in the range of 60–80 °C and 0.12 g of SMA wire were found to produced 2.6 mW of mechanical power and 1.7 mW of electrical power.

## 6. Comparative Analysis of the Power Density

**Figure 27:**
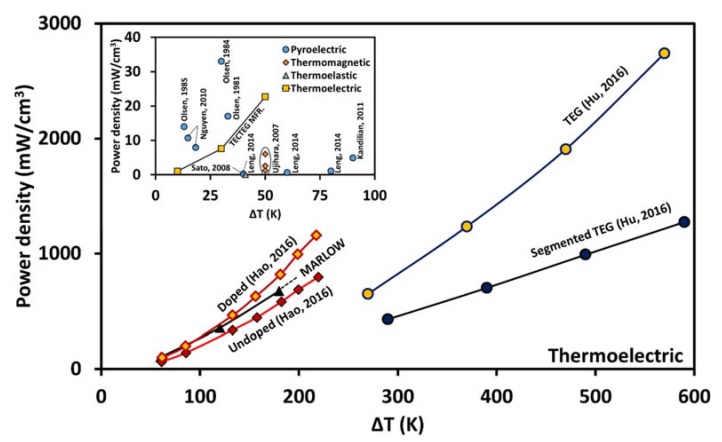
Power density (mW/cm^3^) demonstrated by working prototypes/devices based various thermal energy harvesting technologies. Data are compiled from numerous papers published in the literature and datasheet of commercial products. References: Thermoelectric (TEG2-126LDT, TECTEG MFR. [221]; TG12-2.5-01LS, MARLOW [90]; Undoped and doped BiTe based TEG, Hao et al. 2016 [88]; Non-segmented and segmented nanostructured PbTe based TEG, Hu et al. 2016 [89]), Pyroelectric (PZST based PEG, Oslen et al. 1985 [117]; PZST based regenerative PEG, Oslen et al. 1981 [129]; PZST bases cascaded PEG, Oslen et al. 1984 [128]; P(VDF-TrFE) based PEG, Nguyen et al. 2010 [134]; PVDF film based PEG, Leng et al. 2014 [138]; PMN–32PT single crystal based PEG, Kandilian et al. 2011 [131]), Thermomagnetic (Gadolinium based thermomagnetic harvester, Ujihara et al. 2007 [179]), Thermoelastic (Shape memory alloy heat engine, Sato et al. 2008 [220]).

Figure 27 shows the power density (mW/cm^3^) demonstrated by working prototypes/devices based various thermal energy harvesting technologies reported in literature. These results indicate that thermoelectric generators exhibit very high power density (up to 3.0 kW/cm^3^) at a high temperature regime (temperature difference above 600 K). However, its power density decreases drastically as the temperature difference is decreased. In a low temperature regime, ∆T<100 °C, pyroelectric generators seem to have better power density than thermoelectric generators. Other two technologies, thermomagnetic generators and thermoelastic/SMA engines, have very small power density of less than 1.0 mW/cm^3^.

## 7. Cost Considerations

Cost is a major constraint that hinders the deployment of thermal energy harvesters. The deployment includes costs related to research and development, the working materials, equipment, auxiliary systems, and design services. One of the key factors that influences the deployment cost of thermal energy harvesters is the nature of the heat source. Waste heat is often associated with the by-products of the industrial processes, such as ash and solid waste from coal power plants, slag from steel melting operations, dross from aluminum melters, and bottom waste from reactors. These wastes are often hazardous and need special treatment before they are discharged. Deploying thermal energy harvesters for such applications often requires the advanced equipment and versatile working materials. Most thermal energy harvesters are solid-state devices and thus have low operation and maintenance cost, but the environmental effects such as material corrosion, scaling, and fouling in the heat exchangers cannot be avoided. The deployment cost and the payback period of thermal energy harvesters can be improved with the economy of scale. Unfortunately, thermal energy harvesting, as of now, has been primarily used for small-scale operation, leading to a long payback period.

Thermal energy harvesting is an emerging technology and its economics still need to be analyzed. Most thermal energy harvesters, except TEGs, are not yet commercialized. The cost number for lab prototypes is usually not reported in the literature; therefore, in this review, we have covered only TEGs for cost analysis. Global Thermoelectric Model 8550 by Gentherm Global Power Technologies is possibly the largest commercially TEG system available currently [222]. This system costs approximately $17,500 and generates 550 watt (24 Vdc), resulting in a minimum capital investment (assuming no operating costs or cost of heat source) of about $32/watt [223]. This number seems to be quite high, if we compare it with local electricity pricing. Assuming the average electricity pricing to be $0.08/kWh and a seven-year payback on capital equipment, the capital cost can be calculated to be around $4.9/watt. This implies that the installation of a TEG system in an industrial setting will be favorably received only if the current pricing is reduced by at least seven times. Unarguably, thermal energy harvesting is currently too costly to be adopted in the regular consumer market situation where grid electricity is readily available. This technology requires cost optimization from all fronts to become a commercial reality. A more efficient material, lower device manufacturing costs, and value-driven systems are just a few of the factors to be considered in the further development of this technology.

## 8. Policy Recommendations

Despite the fact that the capital and operating cost for thermal energy harvesting and low-grade heat recovery systems are currently too high, we cannot ignore their positive impact on the environment. Government policies to promote R&D in the area of thermal energy harvesting and the deployment of thermal energy harvesters through subsidized costs can provide a realistic near-term opportunity to improve the nation’s energy infrastructure, environment, and economic future. The list below provides some policy recommendations that would encourage development and deployment of thermal energy harvesters:

- Fund and promote university-based fundamental research projects related to thermal energy harvesting and waste heat recovery.
- Invest in university–industry partnerships to transition the laboratory-based technologies into practice.
- Educate consumers through online and published literature to enhance their awareness about the heat recovery opportunities and its environmental benefits.
- Provide direct financial incentives for each kW of waste heat recovery.
- Offer an investment tax credit on the capital investment related to thermal energy harvesting and heat recovery.
- Offer property tax abatement for facilities that incorporate waste heat recovery.
- Provide low-cost financing to the entities willing to start thermal energy harvesting projects.

## 9. Conclusions

Waste heat and natural heat constitute an enormous energy reserve that can be used to generate electricity in order to fulfil the growing energy demand. The traditional power cycles, such as Rankine and Kalina cycles, are usually not cost-effective, especially for low-grade heat recovery. Thermal energy harvesters are a better option for low-grade waste heat recovery as they are mostly solid-state devices, material-based conversion mechanisms, and require minimal maintenance. This review summarizes the progress made in thermal energy harvesting by improving the figure-of-merit of various material effects: thermoelectricity, pyroelectricity, thermomagneticity, and thermoelasticity. A detailed discussion is provided on governing physics of each mechanism along with device working principles, material performance advancements, and generator prototypes. The effect of controlling parameters that affect the figure of merit of the working materials and the performance of thermal energy harvesters is summarized. A comparison of the power density (power per unit volume) for each of these mechanisms indicated that thermoelectric generators are best suited for applications at temperature difference above 100 °C. At a lower temperature difference, pyroelectric generators were found to be a better option. More specifically, thermoelectric generators were found to exhibit power density up to 3.0 kW/cm^3^ at temperature difference above 600 K. However, its power density decreases drastically as the temperature difference is decreased. When the temperature difference is less than 100 °C, pyroelectric generators have better power density than thermoelectric generators, thermomagnetic generators, and SMA engines. Studying the cost of thermal energy harvesting, it was concluded that the $/W for this technology is currently much higher (about $32/watt) than the local electricity pricing ($4.9/watt). Therefore, cost optimization on all fronts will be required to make these technologies practical. Some policy recommendations to promote thermal energy harvesting and waste heat recovery are also discussed.

## Figures and Tables

**Table 1:** Major waste heat sources and their temperature range [4].

Temp Range	Source	Temp (°C)	Advantages	Disadvantages/Barriers
High >1200 °F (>650 °C)	Nickel refining furnace	1370–1650	High­quality energy Available for a diverse range of end­uses with varying temperature requirements High­efficiency power generation High heat transfer rate per unit area	High temperature creates increased thermal stresses on heat exchange materials Increased chemical activity/corrosion
	Steel electric arc furnace	1370–1650		
	Basic oxygen furnace	1200		
	Aluminum reverberatory furnace	1100–1200		
	Copper refining furnace	760–820		
	Steel heating furnace	930–1040		
	Copper reverberatory furnace	900–1090		
	Hydrogen plants	650–980		
	Fume incinerators	650–1430		
	Glass melting furnace	1300–1540		
	Coke oven	650–1000		
	Iron cupola	820–980		
Medium 450–1200 °F (230–650 °C)	Steam boiler exhaust	230–480	More compatible with heat exchanger materials Practical for power generation	-
	Gas turbine exhaust	370–540		
	Reciprocating engine exhaust	320–590		
	Heat treating furnace	430–650		
	Drying & baking ovens	230–590		
	Cement kiln	450–620		
Low <450 °C (<230 °C)	Exhaust gases exiting recovery devices in gas­fired boilers, ethylene furnaces, etc.	70–230	Large quantities of low temperature heat contained in numerous product streams	Few end uses for low temperature heat Low­efficiency power generation For combustion exhausts, low­temperature heat recovery is impractical due to acidic condensation and heat exchanger corrosion
	Process steam condensate	50–90		
	Cooling water from:	-		
	furnace doors	30–50		
	annealing furnaces	70–230		
	air compressors	30–50		
	internal combustion	70–120		
	engines: air conditioning and air conditioning and	30–40		
	Drying, baking, and curing ovens	90–230		
	Hot processed liquids/solids	30–230		

**Table 2:** Available work output, $W_{cycle}$, based on Olsen cycle for different pyroelectric materials [103]. Data taken from references: [117,122,123,124,125,126,127,128,129,130,131,132,133,134,135,136,137].

Pyroelectric Materials	$T_{c}\;({}^{\circ}C)$	$T_{h}\;({}^{\circ}C)$	$E_{low}\;({\mathrm{MVm}}^{-1})$	$E_{high}\;({\mathrm{MVm}}^{-1})$	$W_{cycle}\;({\mathrm{Jm}}^{-3}/\mathrm{Cycle})$
PNZST ceramic	158	170	0.4	2.8	95
PNZST ceramic	145	175	0.8	3.2	300
PMN-PT 90/10 ceramic	35	85	0.5	3.5	186
PLZT 8/65/35 ceramic	25	160	0.2	7.5	888
KNTM ceramic	140	160	0.1	5	629
BNLT ceramic	25	120	0.1	11.2	1146
BNKT ceramic	25	110	0.1	5.2	1986
BNK-BST ceramic	20	160	0.1	4	1523
YBFO thin	−258	27	0.1	4	7570
PZST	157	177	0.4	3.2	131
PZST	145	178	1.2	3.2	130
PZST	146	159	0	2.9	100
PZST	110	170	0	2.8	0.4
PZN-4.5PT	100	160	0	2	217
PZN-5.5PT	100	190	0	1.2	150
PMN-10PT	30	80	0	3.5	186
PMN-32PT	80	170	0	0.9	100
P(VDF-TrFE) 73/27	23	67	23	53	30
P(VDF-TrFE) 60/40	58	77	4.1	47.2	52
P(VDF-TrFE) 60/40	67	81	20.3	37.9	130
P(VDF-TrFE) 60/40	25	110	20	50	521
P(VDF-TrFE) 60/40	25	120	20	60	900
P(VDF-TrFE-CFE) 61.3/29.7/9	0	25	0	25	50

**Table 3:** Thermoelastic energy conversion efficiency of some of the SMA materials.

Material	$T_{c}\;\left(K\right)$	Δ*T* (K)	$\eta_{absolute}\;(\%)$	$\eta_{rel}\;(\%)$
CuZnAl	293	50	13.5	92.5
CuZnAl	293	80	20.5	95.5
TiNi	323	50	8.6	63.9
TiNi	323	80	15.5	78.2

## References

[1] The Energy Flow Chart Released by Lawrence Livermore National Laboratory. https://flowcharts.llnl.gov/content/assets/images/charts/energy/energy_2011_world.png.

[2] Geothermal Map of the United States. https://www.Nrel.Gov/gis/geothermal.Html.

[3] Johnson I., Choate W.T., Davidson A. (2008). Waste Heat Recovery. Technology and Opportunities in Us Industry.

[4] Thekdi A., Nimbalkar S.U. (2015). Industrial Waste Heat Recovery-Potential Applications, Available Technologies and Crosscutting R&D Opportunities.

[5] Erickson D.C., Anand G., Kyung I. (2004). Heat-activated dual-function absorption cycle. ASHRAE Trans..

[6] Bao J., Zhao L. (2013). A review of working fluid and expander selections for organic rankine cycle. Renew. Sustain. Energy Rev..

[7] Shi L., Shu G., Tian H., Deng S. (2018). A review of modified organic rankine cycles (ORCs) for internal combustion engine waste heat recovery (ICE-WHR). Renew. Sustain. Energy Rev..

[8] Barse K.A., Mann M.D. (2016). Maximizing orc performance with optimal match of working fluid with system design. Appl. Therm. Eng..

[9] Imran M., Usman M., Park B.-S., Kim H.-J., Lee D.-H. (2015). Multi-objective optimization of evaporator of organic rankine cycle (ORC) for low temperature geothermal heat source. Appl. Therm. Eng..

[10] Boyaghchi F.A., Heidarnejad P. (2015). Thermoeconomic assessment and multi objective optimization of a solar micro cchp based on organic rankine cycle for domestic application. Energy Convers. Manag..

[11] Hajabdollahi H., Ganjehkaviri A., Jaafar M.N.M. (2015). Thermo-economic optimization of rsorc (regenerative solar organic rankine cycle) considering hourly analysis. Energy.

[12] Nazari N., Heidarnejad P., Porkhial S. (2016). Multi-objective optimization of a combined steam-organic rankine cycle based on exergy and exergo-economic analysis for waste heat recovery application. Energy Convers. Manag..

[13] Baldi F., Larsen U., Gabrielii C. (2015). Comparison of different procedures for the optimisation of a combined diesel engine and organic rankine cycle system based on ship operational profile. Ocean Eng..

[14] Yang M.-H. (2016). Optimizations of the waste heat recovery system for a large marine diesel engine based on transcritical rankine cycle. Energy.

[15] Tocci L., Pal T., Pesmazoglou I., Franchetti B. (2017). Small scale organic rankine cycle (ORC): A techno-economic review. Energies.

[16] Knudsen T., Clausen L.R., Haglind F., Modi A. (2014). Energy and exergy analysis of the kalina cycle for use in concentrated solar power plants with direct steam generation. Energy Procedia.

[17] Haddad C., Périlhon C., Danlos A., François M.-X., Descombes G. (2014). Some efficient solutions to recover low and medium waste heat: Competitiveness of the thermoacoustic technology. Energy Procedia.

[18] Paanu T., Niemi S., Rantanen P. (2012). Waste Heat Recovery–Bottoming Cycle Alternatives.

[19] Global Geothermal Ltd.. estl.com.au/.

[20] Kalex LLC. http://kalexsystems.com.

[21] Bombarda P., Invernizzi C.M., Pietra C. (2010). Heat recovery from diesel engines: A thermodynamic comparison between kalina and orc cycles. Appl. Therm. Eng..

[22] Radioisotope Thermoelectric Generator (RTG). https://solarsystem.Nasa.Gov/rps/rtg.Cfm.

[23] SAS I. (2012). Ansys Mechanical Apdl Theory Reference.

[24] Hsu C.-T., Huang G.-Y., Chu H.-S., Yu B., Yao D.-J. (2011). An effective seebeck coefficient obtained by experimental results of a thermoelectric generator module. Appl. Energy.

[25] Goldsmid H.J. (2017). Physics of Thermoelectric Energy Conversion.

[26] Nolas G.S., Sharp J., Goldsmid J. (2013). Thermoelectrics: Basic Principles and New Materials Developments.

[27] Choi S.-M., Kim K.-H., Jeong S.-M., Choi H.-S., Lim Y.S., Seo W.-S., Kim I.-H. (2012). A resistance ratio analysis for cosb 3-based thermoelectric unicouples. J. Electron. Microsc..

[28] Wojciechowski K.T., Zybala R., Mania R. (2011). High temperature CoSb_3_-Cu junctions. Microelectron. Reliab..

[29] Lee J.-K., Choi S.-M., Seo W.-S., Lee H.-L., Kim I.-H. (2010). Thermoelectric properties of the co-doped n-type CoSb_3_ compound. J. Korean Phys. Soc..

[30] Song B., Lee S., Cho S., Song M.-J., Choi S.-M., Seo W.-S., Yoon Y., Lee W. (2014). The effects of diffusion barrier layers on the microstructural and electrical properties in cosb 3 thermoelectric modules. J. Alloys Compd..

[31] Key Components of a Thermoelectric Generator (TEG) Module. https://www.Digikey.Co.Nz/en/articles/techzone/2014/apr/thermoelectric-energy-generation-takes-flight-for-aircraft-and-spacecraft-monitoring.

[32] Anant Kishore R., Kumar P., Sanghadasa M., Priya S. (2017). Taguchi optimization of bismuth-telluride based thermoelectric cooler. J. Appl. Phys..

[33] Snyder G.J., Toberer E.S. (2008). Complex thermoelectric materials. Nat. Mater..

[34] Elsheikh M.H., Shnawah D.A., Sabri M.F.M., Said S.B.M., Hassan M.H., Bashir M.B.A., Mohamad M. (2014). A review on thermoelectric renewable energy: Principle parameters that affect their performance. Renew. Sustain. Energy Rev..

[35] Nolas G., Morelli D., Tritt T.M. (1999). Skutterudites: A phonon-glass-electron crystal approach to advanced thermoelectric energy conversion applications. Annu. Rev. Mater. Sci..

[36] Dmitriev A.V., Zvyagin I.P. (2010). Current trends in the physics of thermoelectric materials. Physics-Uspekhi.

[37] Zheng J.-C. (2008). Recent advances on thermoelectric materials. Front. Phys. China.

[38] Yang J., Center G. Designing advanced thermoelectric materials for automotive applications. Proceedings of the DOE/EPRI High Efficiency Thermoelectrics Workshop.

[39] Stabler F.R. (2005). Commercialization of thermoelectric technology. MRS Online Proc. Libr. Arch..

[40] Harman T., Walsh M., Turner G. (2005). Nanostructured thermoelectric materials. J. Electron Microsc..

[41] Venkatasubramanian R., Siivola E., Colpitts T., O’quinn B. (2001). Thin-film thermoelectric devices with high room-temperature figures of merit. Nature.

[42] Hsu K.F., Loo S., Guo F., Chen W., Dyck J.S., Uher C., Hogan T., Polychroniadis E., Kanatzidis M.G. (2004). Cubic AgPb_m_SbTe_2+m_: Bulk thermoelectric materials with high figure of merit. Science.

[43] Rowe D.M. (1995). CRC Handbook of Thermoelectrics.

[44] Morelli D.T., Meisner G.P. (1995). Low temperature properties of the filled skutterudite CeFe_4_Sb_12_. J. Appl. Phys..

[45] Sales B., Mandrus D., Williams R.K. (1996). Filled skutterudite antimonides: A new class of thermoelectric materials. Science.

[46] Nolas G., Slack G., Morelli D., Tritt T., Ehrlich A. (1996). The effect of rare-earth filling on the lattice thermal conductivity of skutterudites. J. Appl. Phys..

[47] Nolas G., Cohn J., Slack G., Schujman S. (1998). Semiconducting Ge clathrates: Promising candidates for thermoelectric applications. Appl. Phys. Lett..

[48] Cohn J., Nolas G., Fessatidis V., Metcalf T., Slack G. (1999). Glasslike heat conduction in high-mobility crystalline semiconductors. Phys. Rev. Lett..

[49] Nolas G.S. (2014). The Physics and Chemistry of Inorganic Clathrates.

[50] Yang R., Chen G. (2005). Nanostructured thermoelectric materials: From superlattices to nanocomposites. Mater. Integr..

[51] Zheng X., Liu C., Yan Y., Wang Q. (2014). A review of thermoelectrics research—Recent developments and potentials for sustainable and renewable energy applications. Renew. Sustain. Energy Rev..

[52] Moyzhes B., Nemchinsky V. (1998). Thermoelectric figure of merit of metal-semiconductor barrier structure based on energy relaxation length. Appl. Phys. Lett..

[53] Shakouri A., Bowers J.E. (1997). Heterostructure integrated thermionic coolers. Appl. Phys. Lett..

[54] Chen G. (2001). Phonon transport in low dimensional. Semicond. Semimet..

[55] Harman T., Taylor P., Walsh M., LaForge B. (2002). Quantum dot superlattice thermoelectric materials and devices. Science.

[56] Poudel B., Hao Q., Ma Y., Lan Y., Minnich A., Yu B., Yan X., Wang D., Muto A., Vashaee D. (2008). High-thermoelectric performance of nanostructured bismuth antimony telluride bulk alloys. Science.

[57] Yim W., Amith A. (1972). Bi-Sb alloys for magneto-thermoelectric and thermomagnetic cooling. Solid-State Electron..

[58] Sidorenko N., Ivanova L. (2001). Bi-Sb solid solutions: Potential materials for high-efficiency thermoelectric cooling to below 180 k. Inorg. Mater..

[59] Gelbstein Y., Dashevsky Z., Dariel M. (2005). High performance n-type pbte-based materials for thermoelectric applications. Phys. B Condens. Matter.

[60] Wu D., Zhao L.-D., Hao S., Jiang Q., Zheng F., Doak J.W., Wu H., Chi H., Gelbstein Y., Uher C. (2014). Origin of the high performance in gete-based thermoelectric materials upon Bi_2_Te_3_ doping. J. Am. Chem. Soc..

[61] Perumal S., Roychowdhury S., Biswas K. (2016). High performance thermoelectric materials and devices based on gete. J. Mater. Chem. C.

[62] Li J., Zhang X., Lin S., Chen Z., Pei Y. (2016). Realizing the high thermoelectric performance of GeTe by Sb-doping and Se-alloying. Chem. Mater..

[63] Zhao L.-D., Zhang X., Wu H., Tan G., Pei Y., Xiao Y., Chang C., Wu D., Chi H., Zheng L. (2016). Enhanced thermoelectric properties in the counter-doped SnTe system with strained endotaxial SrTe. J. Am. Chem. Soc..

[64] Banik A., Shenoy U.S., Saha S., Waghmare U.V., Biswas K. (2016). High power factor and enhanced thermoelectric performance of SnTe-AgInTe_2_: Synergistic effect of resonance level and valence band convergence. J. Am. Chem. Soc..

[65] Zhou M., Gibbs Z.M., Wang H., Han Y., Li L., Snyder G.J. (2016). Thermoelectric performance of co-doped SnTe with resonant levels. Appl. Phys. Lett..

[66] Heikes R.R., Ure R. (1961). Thermoelectricity: Science and Engineering.

[67] Wood C. (1988). Materials for thermoelectric energy conversion. Rep. Prog. Phys..

[68] Ahmad S., Mahanti S., Hoang K., Kanatzidis M. (2006). Ab initio studies of the electronic structure of defects in PbTe. Phys. Rev. B.

[69] Jovovic V., Thiagarajan S., Heremans J., Komissarova T., Khokhlov D., Nicorici A. (2008). Low temperature thermal, thermoelectric, and thermomagnetic transport in indium rich Pb_1−x_Sn_x_Te alloys. J. Appl. Phys..

[70] Heremans J.P., Jovovic V., Toberer E.S., Saramat A., Kurosaki K., Charoenphakdee A., Yamanaka S., Snyder G.J. (2008). Enhancement of thermoelectric efficiency in PbTe by distortion of the electronic density of states. Science.

[71] Snyder G.J., Christensen M., Nishibori E., Caillat T., Brummerstedt Iversen B. (2004). Disordered zinc in Zn_4_Sb_3_ with phonon-glass and electron-crystal thermoelectric properties. Nat. Mater..

[72] Androulakis J., Hsu K.F., Pcionek R., Kong H., Uher C., D’Angelo J.J., Downey A., Hogan T., Kanatzidis M.G. (2006). Nanostructuring and high thermoelectric efficiency in p-Type Ag (Pb_1–y_Sn_y_)_m_SbTe_2+m_. Adv. Mater..

[73] Sootsman J.R., Chung D.Y., Kanatzidis M.G. (2009). New and old concepts in thermoelectric materials. Angew. Chem. Int. Ed..

[74] Wang X.-D., Huang Y.-X., Cheng C.-H., Lin D.T.-W., Kang C.-H. (2012). A three-dimensional numerical modeling of thermoelectric device with consideration of coupling of temperature field and electric potential field. Energy.

[75] Pérez-Aparicio J., Palma R., Taylor R. (2012). Finite element analysis and material sensitivity of peltier thermoelectric cells coolers. Int. J. Heat Mass Transf..

[76] Cheng C.-H., Huang S.-Y., Cheng T.-C. (2010). A three-dimensional theoretical model for predicting transient thermal behavior of thermoelectric coolers. Int. J. Heat Mass Transf..

[77] Meng J.-H., Wang X.-D., Zhang X.-X. (2013). Transient modeling and dynamic characteristics of thermoelectric cooler. Appl. Energy.

[78] Goldsmid H.J. (2010). Introduction to Thermoelectricity.

[79] Ouyang Z., Li D. (2016). Modelling of segmented high-performance thermoelectric generators with effects of thermal radiation, electrical and thermal contact resistances. Sci. Rep..

[80] Kishore R.A., Sanghadasa M., Priya S. (2017). Optimization of segmented thermoelectric generator using taguchi and ANOVA techniques. Sci. Rep..

[81] Kishore R.A., Kumar P., Priya S. (2018). A comprehensive optimization study on Bi_2_Te_3_-based thermoelectric generators using the taguchi method. Sustain. Energy Fuels.

[82] Zebarjadi M., Esfarjani K., Dresselhaus M., Ren Z., Chen G. (2012). Perspectives on thermoelectrics: From fundamentals to device applications. Energy Environ. Sci..

[83] Hu X., Nagase K., Jood P., Ohta M., Yamamoto A. (2015). Power generation evaluated on a bismuth telluride unicouple module. J. Electron. Mater..

[84] Wang H., McCarty R., Salvador J.R., Yamamoto A., König J. (2014). Determination of thermoelectric module efficiency: A survey. J. Electron. Mater..

[85] Wang S., Xie W., Li H., Tang X. (2011). Enhanced performances of melt spun Bi_2_(Te,Se)_3_ for n-type thermoelectric legs. Intermetallics.

[86] Haidar J.G., Ghojel J.I. Waste heat recovery from the exhaust of low-power diesel engine using thermoelectric generators. Proceedings of the 20 International Conference on Thermoelectrics.

[87] Kuroki T., Kabeya K., Makino K., Kajihara T., Kaibe H., Hachiuma H., Matsuno H., Fujibayashi A. (2014). Thermoelectric generation using waste heat in steel works. J. Electron. Mater..

[88] Hao F., Qiu P., Tang Y., Bai S., Xing T., Chu H.-S., Zhang Q., Lu P., Zhang T., Ren D. (2016). High efficiency Bi_2_Te_3_-based materials and devices for thermoelectric power generation between 100 and 300 °C. Energy Environ. Sci..

[89] Hu X., Jood P., Ohta M., Kunii M., Nagase K., Nishiate H., Kanatzidis M.G., Yamamoto A. (2016). Power generation from nanostructured pbte-based thermoelectrics: Comprehensive development from materials to modules. Energy Environ. Sci..

[90] TG12-4 TEG. https://www.Marlow.Com.

[91] ThermaWatt, a Candle Powered TEG. http://www.Tegmart.Com/diy-teg-kits/diy-candle-powered-teg-with-led-options/.

[92] DW-DF-10W Camp Stove TEG. http://www.Tegpower.Com/pro2.Htm.

[93] EverGen PowerStrap. http://www.Marlow.Com/power-generators/evergen-power-strap.Html.

[94] MPG-D655, Micropelt Thermogenerator Chip. http://www.Micropelt.Com.

[95] Green Car Congress. http://www.Greencarcongress.Com/2011/08/bmwthermal-20110830.Html.

[96] Lang S.B. (2005). Pyroelectricity: From ancient curiosity to modern imaging tool. Phys. Today.

[97] Donnay G. (1977). Structural mechanism of pyroelectricity in tourmaline. Acta Crystallogr. Sect. A.

[98] Hawkins K.D., Mackinnon I.D., Schneeberger H. (1995). Influence of chemistry on the pyroelectric effect in tourmaline. Am. Miner..

[99] Shur M., Bykhovski A., Gaska R. (1998). Pyroelectric and piezoelectric properties of gan-based materials. MRS Online Proc. Libr. Arch..

[100] Zhang J., Wang C. (2016). Size-dependent pyroelectric properties of gallium nitride nanowires. J. Appl. Phys..

[101] Hunter S.R., Lavrik N.V., Mostafa S., Rajic S., Datskos P.G. (2012). Review of Pyroelectric Thermal Energy Harvesting and New Mems-Based Resonant Energy Conversion Techniques. Proc. SPIE.

[102] Guyomar D., Sebald G. (2009). Pyroelectric/electrocaloric energy scanvenging and cooling capabilities in ferroelectric materials. Int. J. Appl. Electromagn. Mech..

[103] Bowen C.R., Taylor J., LeBoulbar E., Zabek D., Chauhan A., Vaish R. (2014). Pyroelectric materials and devices for energy harvesting applications. Energy Environ. Sci..

[104] Cuadras A., Gasulla M., Ferrari V. (2010). Thermal energy harvesting through pyroelectricity. Sensors Actuators A Phys..

[105] Mane P., Xie J., Leang K.K., Mossi K. (2011). Cyclic energy harvesting from pyroelectric materials. IEEE Trans. Ultrason. Ferroelectr. Freq. Control.

[106] Whatmore R. (1986). Pyroelectric devices and materials. Rep. Prog. Phys..

[107] Whatmore R.W., Watton R. (2001). Pyroelectric materials and devices. Infrared Detectors and Emitters: Materials and Devices.

[108] Li X., Lu S.-G., Chen X.-Z., Gu H., Qian X.-S., Zhang Q. (2013). Pyroelectric and electrocaloric materials. J. Mater. Chem. C.

[109] Cooper J. (1962). A fast-response pyroelectric thermal detector. J. Sci. Instrum..

[110] Ivill M., Ngo E., Cole M.W. (2013). Method and Characterization of Pyroelectric Coefficients for Determining Material Figures of Merit for Infrared (IR) Detectors.

[111] Noh J.Y., Yoon G.H. (2012). Topology optimization of piezoelectric energy harvesting devices considering static and harmonic dynamic loads. Adv. Eng. Softw..

[112] Alpay S.P., Mantese J., Trolier-McKinstry S., Zhang Q., Whatmore R.W. (2014). Next-generation electrocaloric and pyroelectric materials for solid-state electrothermal energy interconversion. MRS Bull..

[113] Childress J. (1962). Application of a ferroelectric material in an energy conversion device. J. Appl. Phys..

[114] Hoh S. (1963). Conversion of thermal to electrical energy with ferroelectric materials. Proc. IEEE.

[115] Clingman W., Moore R. (1961). Application of ferroelectricity to energy conversion processes. J. Appl. Phys..

[116] Sebald G., Lefeuvre E., Guyomar D. (2008). Pyroelectric energy conversion: Optimization principles. IEEE Trans. Ultrason. Ferroelectr. Freq. Control.

[117] Olsen R.B., Bruno D.A., Briscoe J.M. (1985). Pyroelectric conversion cycles. J. Appl. Phys..

[118] Frood D.G. (1954). A note on the use of the titanates as thermoelectric transducers. Can. J. Phys..

[119] Drummond J. Dielectric power conversion. Proceedings of the Annual Intersociety Energy Conversion and Engineering Conference.

[120] Olsen R., Brown D. (1982). High efficieincy direct conversion of heat to electrical energy-related pyroelectric measurements. Ferroelectrics.

[121] Olsen R.B., Evans D. (1983). Pyroelectric energy conversion: Hysteresis loss and temperature sensitivity of a ferroelectric material. J. Appl. Phys..

[122] Sebald G., Pruvost S., Guyomar D. (2007). Energy harvesting based on ericsson pyroelectric cycles in a relaxor ferroelectric ceramic. Smart Mater. Struct..

[123] Lee F.Y., Goljahi S., McKinley I.M., Lynch C.S., Pilon L. (2012). Pyroelectric waste heat energy harvesting using relaxor ferroelectric 8/65/35 plzt and the olsen cycle. Smart Mater. Struct..

[124] Vats G., Chauhan A., Vaish R. (2015). Thermal energy harvesting using bulk lead-free ferroelectric ceramics. Int. J. Appl. Ceram. Technol..

[125] Chauhan A., Patel S., Vats G., Vaish R. (2014). Enhanced thermal energy harvesting using Li, K-Doped Bi_0.5_Na_0.5_TiO_3_ lead-free ferroelectric ceramics. Energy Technol..

[126] Vats G., Vaish R., Bowen C.R. (2014). An analysis of lead-free (Bi_0.5_Na_0.5_)_0.915_-(Bi_0.5_K_0.5_)_0.05_Ba_0.02_Sr_0.015_TiO_3_ ceramic for efficient refrigeration and thermal energy harvesting. J. Appl. Phys..

[127] Vats G., Kushwaha H.S., Vaish R. (2014). Enormous energy harvesting and storage potential in multiferroic epitaxial thin film hetrostructures: An unforeseen era. Mater. Res. Express.

[128] Olsen R., Bruno D., Briscoe J., Dullea J. (1984). Cascaded pyroelectric energy converter. Ferroelectrics.

[129] Olsen R.B., Briscoe J.M., Bruno D.A., Butler W.F. (1981). A pyroelectric energy converter which employs regeneration. Ferroelectrics.

[130] Khodayari A., Pruvost S., Sebald G., Guyomar D., Mohammadi S. (2009). Nonlinear pyroelectric energy harvesting from relaxor single crystals. IEEE Trans. Ultrason. Ferroelectr. Freq. Control.

[131] Kandilian R., Navid A., Pilon L. (2011). The pyroelectric energy harvesting capabilities of PMN–PT near the morphotropic phase boundary. Smart Mater. Struct..

[132] Olsen R.B., Bruno D.A., Briscoe J.M., Jacobs E.W. (1985). Pyroelectric conversion cycle of vinylidene fluoride-trifluoroethylene copolymer. J. Appl. Phys..

[133] Ikura M. (2002). Conversion of low-grade heat to electricity using pyroelectric copolymer. Ferroelectrics.

[134] Nguyen H., Navid A., Pilon L. (2010). Pyroelectric energy converter using co-polymer P (VDF-TrFE) and olsen cycle for waste heat energy harvesting. Appl. Therm. Eng..

[135] Navid A., Pilon L. (2011). Pyroelectric energy harvesting using olsen cycles in purified and porous poly (vinylidene fluoride-trifluoroethylene) [P (VDF-TrFE)] thin films. Smart Mater. Struct..

[136] Olsen R., Bruno D. Pyroelectric conversion materials. Proceedings of the Twenty-First Intersociety Energy Conversion Engineering Conference.

[137] Zhu H., Pruvost S., Cottinet P., Guyomar D. (2011). Energy harvesting by nonlinear capacitance variation for a relaxor ferroelectric poly (vinylidene fluoride-trifluoroethylene-chlorofluoroethylene) terpolymer. Appl. Phys. Lett..

[138] Leng Q., Chen L., Guo H., Liu J., Liu G., Hu C., Xi Y. (2014). Harvesting heat energy from hot/cold water with a pyroelectric generator. J. Mater. Chem. A.

[139] Olsen R.B. (1982). Ferroelectric conversion of heat to electrical energy—A demonstration. J. Energy.

[140] Yang Y., Wang S., Zhang Y., Wang Z.L. (2012). Pyroelectric nanogenerators for driving wireless sensors. Nano Lett..

[141] Lee J.H., Lee K.Y., Gupta M.K., Kim T.Y., Lee D.Y., Oh J., Ryu C., Yoo W.J., Kang C.Y., Yoon S.J. (2014). Highly stretchable piezoelectric-pyroelectric hybrid nanogenerator. Adv. Mater..

[142] Kishore R.A., Priya S. (2018). A review on design and performance of thermomagnetic devices. Renew. Sustain. Energy Rev..

[143] Solomon D. (1988). Improving the performance of a thermomagnetic generator by cycling the magnetic field. J. Appl. Phys..

[144] Joshi K.B., Priya S. (2013). Multi-physics model of a thermo-magnetic energy harvester. Smart Mater. Struct..

[145] Solomon D. (1991). Design of a thermomagnetic generator. Energy Convers. Manag..

[146] Kishore R.A., Priya S. (2017). Low-grade waste heat recovery using reverse magnetocaloric effect. Sustain. Energy Fuels.

[147] Hsu C.-J., Sandoval S.M., Wetzlar K.P., Carman G.P. (2011). Thermomagnetic conversion efficiencies for ferromagnetic materials. J. Appl. Phys..

[148] Post A., Knight C., Kisi E. (2013). Thermomagnetic energy harvesting with first order phase change materials. J. Appl. Phys..

[149] Elliott J. (1959). Thermomagnetic generator. J. Appl. Phys..

[150] Kirol L.D., Mills J.I. (1984). Numerical analysis of thermomagnetic generators. J. Appl. Phys..

[151] Phan M.-H., Peng H.-X., Yu S.-C., Tho N.D., Nhat H.N., Chau N. (2007). Manganese perovskites for room temperature magnetic refrigeration applications. J. Magn. Magn. Mater..

[152] Srivastava V., Song Y., Bhatti K., James R. (2011). The direct conversion of heat to electricity using multiferroic alloys. Adv. Energy Mater..

[153] Song Y. (2014). Performance analysis of energy conversion via caloric effects in first-order ferroic phase transformations. Phys. Chem. Chem. Phys..

[154] Song Y., Bhatti K.P., Srivastava V., Leighton C., James R.D. (2013). Thermodynamics of energy conversion via first order phase transformation in low hysteresis magnetic materials. Energy Environ. Sci..

[155] Pecharsky V.K., Gschneidner K.A. (1997). Giant magnetocaloric effect in Gd_5_(Si_2_Ge_2_). Phys. Rev. Lett..

[156] Klimczak M., Talik E. (2010). In Magnetocaloric effect of GdTX (T = Mn, Fe, Ni, Pd, X = Al, In) and GdFe_6_Al_6_ ternary compounds. J. Phys. Conf. Ser..

[157] Law J., Ramanujan R., Franco V. (2010). Tunable Curie temperatures in Gd alloyed Fe–B–Cr magnetocaloric materials. J. Alloy. Compd..

[158] Canepa F., Napoletano M., Cirafici S. (2002). Magnetocaloric effect in the intermetallic compound Gd_7_Pd_3_. Intermetallics.

[159] Spichkin Y., Pecharsky V., Gschneidner K. (2001). Preparation, crystal structure, magnetic and magnetothermal properties of (Gd_x_R_5−x_)Si_4_, where R = Pr and Tb, alloys. J. Appl. Phys..

[160] Von Ranke P., De Oliveira N., Gama S. (2004). Theoretical investigations on giant magnetocaloric effect in MnAs_1−x_Sb_x_. Phys. Lett. A.

[161] Wada H., Morikawa T., Taniguchi K., Shibata T., Yamada Y., Akishige Y. (2003). Giant magnetocaloric effect of MnAs_1−x_Sb_x_ in the vicinity of first-order magnetic transition. Phys. B Condens. Matter.

[162] Wada H., Taniguchi K., Tanabe Y. (2002). Extremely large magnetic entropy change of MnAs_1−x_Sb_x_ near room temperature. Mater. Trans..

[163] Das S., Dey T. (2007). Magnetic entropy change in polycrystalline La_1−x_K_x_ MnO_3_ perovskites. J. Alloy. Compd..

[164] Zhong W., Chen W., Au C., Du Y. (2003). Dependence of the magnetocaloric effect on oxygen stoichiometry in polycrystalline La_2/3_Ba_1/3_MnO_3–δ_. J. Magn. Magn. Mater..

[165] Hanh D., Islam M., Khan F., Minh D., Chau N. (2007). Large magnetocaloric effect around room temperature in La_0.7_Ca_0.3−x_Pb_x_MnO_3_ perovskites. J. Magn. Magn. Mater..

[166] Phan M.-H., Tian S.-B., Yu S.-C., Ulyanov A. (2003). Magnetic and magnetocaloric properties of La_0.7_Ba_0.3−x_Pb_x_MnO_3_ compounds. J. Magn. Magn. Mater..

[167] Sun W., Li J., Ao W., Tang J., Gong X. (2006). Hydrothermal synthesis and magnetocaloric effect of La_0.7_Ca_0.2_Sr_0.1_MnO_3_. Powder Technol..

[168] Li J., Sun W., Ao W., Tang J. (2006). Hydrothermal synthesis and magnetocaloric effect of La_0.5_Ca_0.3_Sr_0.2_MnO_3_. J. Magn. Magn. Mater..

[169] Tesla N. (1889). Thermo-Magnetic Motor. U.S. Patent.

[170] Edison T.A. (1888). Pyromagnetic Motor. U.S. Patent.

[171] Tesla N. (1890). Pyromagneto-Electric Generator. U.S. Patent.

[172] Edison T.A. (1892). Pyromagnetic Generator. U.S. Patent.

[173] Van der Maas G.J., Purvis W.J. (1956). “Curie point” motor. Am. J. Phys..

[174] Murakami K., Nemoto M. (1972). Some experiments and considerations on the behavior of thermomagnetic motors. IEEE Trans. Magn..

[175] Takahashi Y., Matsuzawa T., Nishikawa M. (2004). Fundamental performance of the disc-type thermomagnetic engine. Electr. Eng. Jpn..

[176] Takahashi Y., Yamamoto K., Nishikawa M. (2006). Fundamental performance of triple magnetic circuit type cylindrical thermomagnetic engine. Electr. Eng. Jpn..

[177] Palmy C. (2006). A new thermo-magnetic wheel. Eur. J. Phys..

[178] Palmy C. (2007). A thermo-magnetic wheel. Eur. News.

[179] Ujihara M., Carman G., Lee D. (2007). Thermal energy harvesting device using ferromagnetic materials. Appl. Phys. Lett..

[180] Chun J., Song H.-C., Kang M.-G., Kang H.B., Kishore R.A., Priya S. (2017). Thermo-magneto-electric generator arrays for active heat recovery system. Sci. Rep..

[181] Massad J.E., Smith R.C. (2005). A homogenized free energy model for hysteresis in thin-film shape memory alloys. Thin Solid Films.

[182] Müller I., Xu H. (1991). On the pseudo-elastic hysteresis. Acta Met. Mater..

[183] Fedelich B., Zanzotto G. (1991). One-dimensional quasistatic nonisothermal evolution of shape-memory material inside the hysteresis loop. Contin. Mech. Thermodyn..

[184] Raniecki B., Lexcellent C., Tanaka K. (1992). Thermodynamic models of pseudoelastic behaviour of shape memory alloys. Arch. Mech..

[185] Tanaka K., Hayashi T., Itoh Y., Tobushi H. (1992). Analysis of thermomechanical behavior of shape memory alloys. Mech. Mater..

[186] Ziótkowski A. (1993). Theoretical analysis of efficiency of shape memory alloy heat engines (based on constitutive models of pseudoelasticity). Mech. Mater..

[187] Qian S., Ling J., Hwang Y., Radermacher R., Takeuchi I. (2015). Thermodynamics cycle analysis and numerical modeling of thermoelastic cooling systems. Int. J. Refrig..

[188] Lagoudas D.C. (2008). Shape Memory Alloys: Modeling and Engineering Applications.

[189] Kurdjumov G., Khandros L. (1949). First reports of the thermoelastic behaviour of the martensitic phase of Au-Cd alloys. Dokl. Akad. Nauk SSSR.

[190] Buehler W.J., Gilfrich J., Wiley R. (1963). Effect of low-temperature phase changes on the mechanical properties of alloys near composition tini. J. Appl. Phys..

[191] Stöckel D. (1995). The shape memory effect-phenomenon, alloys and applications. California.

[192] Elahinia M.H., Hashemi M., Tabesh M., Bhaduri S.B. (2012). Manufacturing and processing of niti implants: A review. Prog. Mater. Sci..

[193] Melton K., Mercier O. (1978). Deformation behavior of NiTi-based alloys. Met. Trans. A.

[194] Miyazaki S., Mizukoshi K., Ueki T., Sakuma T., Liu Y. (1999). Fatigue life of Ti–50 at.% Ni and Ti–40Ni–10Cu (at.%) shape memory alloy wires. Mater. Sci. Eng. A.

[195] Doonkersloot H., Vucht V. (1970). Martensitic transformations in Au-Ti, Pd-Ti and Pt-Ti alloys. J. Less-Common Met..

[196] Otsuka K., Wayman C.M. (1999). Shape Memory Materials.

[197] Zhu J., Liang N., Liew K., Huang W. (2001). Energy conversion in shape memory alloy heat engine part i: Theory. J. Intell. Mater. Syst. Struct..

[198] Funakubo H., Kennedy J. (1987). Shape Memory Alloys.

[199] Shin M.C., Kim C.S., Chung Y.H., Jee K.K. (1987). Twin-Crank Type Heat Engine. U.S. Patent.

[200] Banks R. (1986). Vertically Oscillating Heat Engine. U.S. Patent.

[201] Wechsler M.S., Van Gerpen J.H. (1994). Apparatus for Recovery and Use of Waste Thermal Energy. U.S. Patent.

[202] Iwanaga H., Tobushi H., Ito H. (1988). Basic research on output power characteristics of a shape memory alloy heat engine:(twin crank heat engine). JSME.

[203] Sandoval D.J. (1977). Thermal Motor. U.S. Patent.

[204] Wang F.E. (1981). Energy Conversion System. U.S. Patent.

[205] Pachter J.J. (1979). Engine. U.S. Patent.

[206] Wayman C.M. (1981). Solid State Thermal Engine. U.S. Patent.

[207] Cory J.S. (1981). Solid State Heat Engine. U.S. Patent.

[208] Cory J.S. (1977). Variable Density Heat Engine. U.S. Patent.

[209] Johnson A.D., Kirkpatrick P.F. (1981). Field Effect Memory Alloy Heat Engine. U.S. Patent.

[210] Dillon C.L. (1984). Engine Construction. U.S. Patent.

[211] O’hare L.R. (1987). Wire Engine. U.S. Patent.

[212] Ahlers M. (1975). On the usefulness of martensitic transformations for energy conversion. Scr. Met..

[213] Golestaneh A. (1978). Efficiency of the solid-state engine made with nitinol memory material. J. Appl. Phys..

[214] Mogutnov B. (1978). The efficiency of heat engines having a solid working body based on alloys with a shape’memory’ effect. Fiz. Met. Met..

[215] Wu W.-F., Long X.-P., Yu X.-L., Feng Q.-K. (2012). A new power generation method utilizing a low grade heat source. J. Zhejiang Univ. Sci. A.

[216] Salzbrenner R. (1984). Shape memory heat engines. J. Mater. Sci..

[217] Schiller E.H. (2002). Heat Engine Driven by Shape Memory Alloys: Prototyping and Design. Master’s Thesis.

[218] Wakjira J.F. (2001). The vt1 Shape Memory Alloy Heat Engine Design. Master’s Thesis.

[219] Avirovik D., Kumar A., Bodnar R.J., Priya S. (2013). Remote light energy harvesting and actuation using shape memory alloy-Piezoelectric hybrid transducer. Smart Mater. Struct..

[220] Sato Y., Yoshida N., Tanabe Y., Fujita H., Ooiwa N. (2008). Characteristics of a new power generation system with application of a shape memory alloy engine. Electr. Eng. Jpn..

[221] Avirovik D., Kishore R.A., Vuckovic D., Priya S. (2014). Miniature shape memory alloy heat engine for powering wireless sensor nodes. Energy Harvest. Syst..

[222] Gentherm Global Power Technologies. http://www.Genthermglobalpower.Com/products/thermoelectric-generators-tegs.

[223] Hendricks T., Choate W.T. (2006). Engineering Scoping Study of Thermoelectric Generator Systems for Industrial Waste Heat Recovery.

